# Recognition of non-CpG repeats in Alu and ribosomal RNAs by the Z-RNA binding domain of ADAR1 induces A-Z junctions

**DOI:** 10.1038/s41467-021-21039-0

**Published:** 2021-02-04

**Authors:** Parker J. Nichols, Shaun Bevers, Morkos Henen, Jeffrey S. Kieft, Quentin Vicens, Beat Vögeli

**Affiliations:** 1 Department of Biochemistry and Molecular Genetics, University of Colorado Denver School of Medicine, Aurora, CO 80045 USA; 2 Colorado School of Mines, Golden, CO 80401 USA; 3 Faculty of Pharmacy, Mansoura University, Mansoura, 35516 Egypt; 4 RNA BioScience Initiative, University of Colorado Denver School of Medicine, Aurora, CO 80045 USA

**Keywords:** Molecular biophysics, RNA, NMR spectroscopy

## Abstract

Adenosine-to-inosine (A-to-I) editing of eukaryotic cellular RNAs is essential for protection against auto-immune disorders. Editing is carried out by ADAR1, whose innate immune response-specific cytoplasmic isoform possesses a Z-DNA binding domain (Zα) of unknown function. Zα also binds to CpG repeats in RNA, which are a hallmark of Z-RNA formation. Unexpectedly, Zα has been predicted — and in some cases even shown — to bind to specific regions within mRNA and rRNA devoid of such repeats. Here, we use NMR, circular dichroism, and other biophysical approaches to demonstrate and characterize the binding of Zα to mRNA and rRNA fragments. Our results reveal a broad range of RNA sequences that bind to Zα and adopt Z-RNA conformations. Binding is accompanied by destabilization of neighboring A-form regions which is similar in character to what has been observed for B-Z-DNA junctions. The binding of Zα to non-CpG sequences is specific, cooperative and occurs with an affinity in the low micromolar range. This work allows us to propose a model for how Zα could influence the RNA binding specificity of ADAR1.

## Abstract (web summary)

ADAR1 is an interferon-induced enzyme that catalyzes editing of adenine to inosine across the transcriptome as part of the immune response. Here the authors establish how ADAR1 recognizes non-CpG RNA sequences to facilitate the formation of A-Z junctions.

## Introduction

Distinguishing between self and non-self RNA is a hallmark of the innate immune system. In humans, self RNAs are edited by an adenosine deaminase that acts on RNA (ADAR1), which converts some adenosines to inosines^1–3^. Because inosines tend to disrupt double-stranded regions, such RNAs are not recognized by PKR, MDA5, and RIG-I, which are enzymes that trigger an immune response through binding to foreign and therefore unedited double-stranded regions^4,5^.

**Fig. 1:**
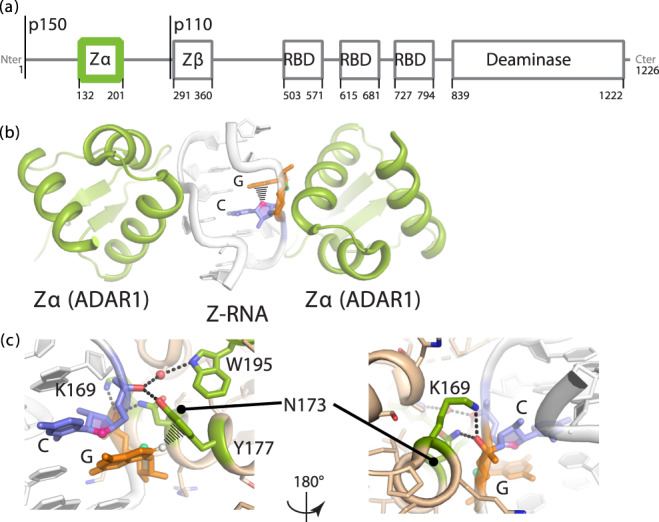
The Zα domain of ADAR1p150 binds to Z-RNA. **a** Domain organization of ADAR1: Zα and Zβ are structurally homologous helix-turn-helix DNA-binding domains, RBD stands for double-stranded RNA binding domain. Both isoforms are indicated. **b** Crystal structure of (CpG)_3_ bound to Zα from ADAR1 (PDB ID: 2GXB)^25^. **c** Close-up of the RNA-protein interface.

ADAR1 is constitutively expressed in most cells as a stable p110 isoform localized in the nucleus^6–8^. Upon invasion by a pathogen, the cell launches an interferon (IFN) response, resulting in the expression of a longer p150 isoform, which contributes to resisting the infection by editing self RNAs in the cytoplasm^6,9^ (Fig. 1a). A-to-I editing is therefore augmented during the IFN response, primarily through the action of ADAR1p150^4^.

Although infections lead to a massive increase in editing events, A-to-I changes do not occur nonspecifically. Approximately 90% of A-to-I editing events occur at primate-specific Alu elements^10–12^, the most ubiquitous family of short-interspersed repeats within the human genome^13^. Alu elements recently have been shown to be one of the primary activators of IFN in relapsing-remitting multiple sclerosis^14^ and enrichment of Alu RNAs may be a common factor of autoimmune diseases^15,16^. These observations may directly relate to recent findings that host Alu RNAs are one of the primary activators of RIG-I during Kaposi’s sarcoma-associated herpesvirus infection^17,18^. This suggests that an essential role of ADAR1—and ADAR1p150 in particular—is to edit Alu elements so that activation of dsRNA sensors is prevented. ADAR1 is therefore part of the strategy to modulate the delicate balance between turning the IFN response on or off.

ADAR1p150 catalyzes A-to-I editing within Alu “foldback” structures, where an Alu element pairs with an inverted Alu element located a few hundred nucleotides away on the same RNA^4,19^. How ADAR1p150 achieves this level of specificity is unknown. Part of the answer likely lies within its N-terminus, which comprises a Zα and a Zβ domain separated by a linker of ~100 amino acids. While Zβ is present in both isoforms, Zα is present only in the IFN-induced ADAR1p150. Zα is a member of a family of helix-turn-helix domains that recognize the unusual geometry of the Z-conformation in DNA or RNA and bind to five base pairs in a symmetrical fashion (Fig. 1b)^20,21^. Based on the structural similarity between Z-DNA and Z-RNA, it was proposed and verified that Zα binds to CpG-only RNAs with a dissociation constant (K_d_) of ~9 nM^22,23^. Furthermore, the addition of CpG repeat sequences to ADAR1p150 substrates significantly increases the deamination of neighboring adenosines^24^. Whether Zα also binds to less idealized sequences, with what K_d_, and following what binding process are fundamental questions that remain to be answered.

The crystal structure of Zα in complex with a (CpG)_3_ RNA duplex showed that binding specificity is achieved through a critical tyrosine residue (Tyr177), which makes a C–H…π interaction with the *syn* purine in the CpG Z-step (Fig. 1c)^25,26^. Tyr177 is positioned to interact with Z-RNA through a network of interactions involving other amino acids (notably Lys169, Asn173, and Trp195; Fig. 1c), which cause a 60% reduction in A-to-I editing levels when they are mutated (^5,27,28^). This reduction in editing leads to the Aicardi-Goutières syndrome^28^, a neurodevelopmental disorder characterized by hyperactive immune responses. Zα is clearly important for the integrity of the editing process, but how does that relate to its ability to bind Z-DNA and/or Z-RNA?

Although most studies of Zα were done with CpG repeats, Zα can also recognize TpA, CpA, GpC, and TpG in DNA^20,29^. This observation indicates that many dinucleotide sequences can adopt a Z-conformation, as observed in RNA for an even broader range of sequences (Supplementary Fig. 1)^30^. Although non-CpG sequences within DNA bind to Zα, the resulting complexes are less stable compared to CpG repeats^23^. Intriguingly, the adoption of Z-conformations within the context of larger dsDNA stretches requires the formation of B-Z junctions, where the B-DNA regions flanking the Z-forming sequence become destabilized by Zα binding, and nucleotides adjacent to the Z-DNA sequence flip out in order to create continuous base-stacking between the B- and Z-DNA^31–34^. In such conformations, four Zα molecules are bound to an eight base-pair stretch, indicating that Zα can bind to a variable number of base pairs depending on the context^31^. While the formation of equivalent A-Z junctions in RNA has been shown to be possible by fluorescence studies^35^, our knowledge of the sequence preferences and contexts for the formation of such conformations upon Zα binding is limited. Within RNA, some of the sequence combinations (especially UpG and UpA repeats) have been predicted to shift to Z-RNA within the heavily edited Alu foldback structures^36^. In further support of a broad recognition of RNA sequences by Zα, pull-down assays revealed that Zα binds to ribosomal stem-loops sometimes devoid of CpG repeats^37^. This broader RNA sequence specificity is in support of the Zα-mediated increase in ADAR1’s specificity and activity proposed by Rich^24^, and could also explain the recently described surge in mouse anti-viral response upon sensing endogenous retroviral elements by the Zα domain of Z-DNA-binding protein 1^38^.

We hypothesize that the >10,000 editing sites in the human transcriptome^11,12^ are associated with widespread Z-RNA formation at CpG as well as non-CpG sequences within Alu foldbacks. To begin to address this possibility, we applied NMR, circular dichroism (CD), isothermal titration calorimetry (ITC), and analytical ultracentrifugation (AUC) to characterize the binding of Zα to RNA fragments with various frequencies of CpG repeats. We demonstrate binding of Zα at specific sites on a fragment from an Alu foldback rich in UpG steps (AluSx1Jo;^36^) and on hairpins from bacterial and human ribosomal RNAs that contain various types of YpR repeats (Y = pyrimidine, R = purine)^37^. Our NMR results show that binding results in the formation of A-Z junctions in these RNAs and that RNAs with regions of low helical stability adjacent to a Z-forming sequence are better Z-RNA adopters. For the Alu fragment and for the *E. coli* h43 hairpin in particular, we further determine that two Zα molecules bind every four to five base pairs, and that binding is cooperative, with K_D_ values of 1–9 µM and 40–110 nM. In sum, when binding to AluSx1Jo, Zα adopts a conformation that has characteristics of both a B-Z DNA junction and the (CpG)_3_ RNA repeat. Overall, our work offers a rationale for earlier observations that ADAR1 binds in multiple steps to double-stranded RNA regions often flanked by regions rich in non-Watson–Crick pairs^39,40^. This study also provides a framework for better understanding widespread and pervasive editing in cells.

## Results

### Zα induces A-Z junctions within an AluSx1Jo foldback fragment and ribosomal hairpin h43

**Fig. 2:**
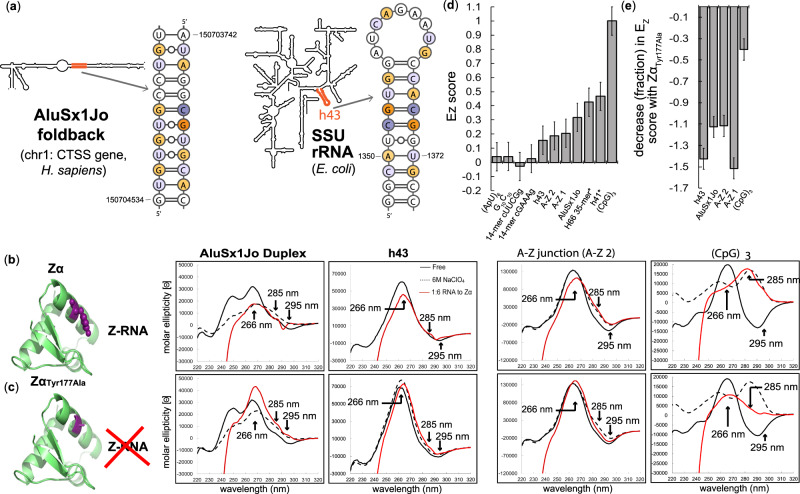
Zα induces a partial Z-conformation in AluSx1Jo and h43. **a** Location of AluSx1Jo and h43 on secondary structure diagrams of the *CTSS* gene (Chromosome 1) from *H. sapiens* and the small subunit ribosomal RNA from *E. coli*. For h43, the first three G = C base pairs were engineered for added stability. In this and subsequent figures, CpG and YpR steps are shown in dark (CpG) and light (non-CpG YpR) shades of purple/orange colors. **b** CD spectra of the AluSx1Jo and h43 RNAs in absence of protein (black) and at the 1:6 RNA:Zα ratio (red), at which binding is saturated. Controls: 6 M NaClO_4_ (dotted line), ionic condition that promotes Z-RNA formation of CpG repeats^57^; A-Z junction^35^ (second to right-most panel), which is an RNA that has a (CpG)_6_ sequence followed by an A-RNA forming sequence, and (CpG)_3_ (right-most panel), which is fully converted to Z-RNA at a 1:2 RNA:Zα ratio. **c** Same as **b**, but with Zα_Tyr177Ala_ instead of wild-type Zα. **d** *E*_*Z*_ scores quantifying the extent of Z-conformation for the following fragments: (ApU)_6_, G_10_C_10_, 14mer cUUCGg, cGAAAg tetraloop (negative controls); h43 from *E. coli*; AluSx1Jo; H66 35mer from *H. sapiens*; (CpG)_3_ (positive control). **e** Reduction in *E*_*Z*_ score for AluSx1Jo, h43, and (CpG)3 RNAs (expressed as a fraction) when Zα_Tyr177Ala_ is used instead of Zα. * indicates that RNA forms a duplex as determined by AUC when stem-loop was expected (Supplementary Fig. 5). An error of 0.1 was determined to be appropriate for the calculated *E*_*Z*_ scores by taking into account the difference between the (CpG)_3_ and (CpG)_6_ RNAs (which theoretically both have an *E*_*Z*_ score of 1) and the difference in the *E*_*Z*_ score between repeat measurements on h43 *E.coli*. All other *E*_*Z*_ scores were determined from one CD measurement.

The core model systems of our study are an element from the AluSx1Jo foldback within the *Cathepsin S (CTSS)* gene on human chromosome 1, as well as hairpin 43 from the small subunit of the *E. coli* ribosome comprising hairpin h43 (Fig. 2a). This region of AluSx1Jo had been predicted to adopt a Z-conformation^36^, and h43 had been reported to bind to Zα in vitro^37^. The AluSx1Jo region comprises predominantly UpG repeats, but also one CpG, one CpA, and one UpA dinucleotide steps. Hairpin h43 contains a symmetrical (or ‘canonical’) CpG step together with several UpG and CpA steps. Based on current knowledge of Zα recognition, it is not immediately apparent how these two RNAs could adopt the Z-RNA conformation required for Zα binding.

Using chemically synthesized RNA fragments for AluSx1Jo, h43, and the control sequences (CpG)_3_ and two A-Z junctions, we first carried out CD experiments, which have traditionally been used to monitor transitions to the Z-conformation in DNA and RNA^22,41^. Upon titrating increasing amounts of Zα to the control (CpG)_3_ fragment (from 12:1 to 1:6 RNA:Zα), we observed the characteristic decrease in the A-form peak at 266 nm, the appearance of a peak at 285 nm, and the flip from negative to positive at 295 nm (Fig. 2b, full titration shown in Supplementary Fig. 2). Together, these three changes were indicative of a conversion to the Z-conformation^22^, and are unlikely to be due to a conformational change in Zα upon binding RNA as our ^15^N-HSQC titration indicates binding only results in minor rearrangements to the RNA binding residues of Zα (Supplemental Figs. S13 and S14). However, our selected test RNAs had diverse sequence context and likely did not fully adopt the Z-conformation. Performing the same experiment with RNAs which form A-Z junctions (A-Z 1 is the RNA version of a previously characterized B-Z junction^31^, and A-Z 2 has been shown by fluorescence studies to adopt an A-Z junction^35^), we observed instead a reduction of the peak at 266 nm and smaller-in-magnitude increases to the molar ellipticities values at 285 and 295 nm (Fig. 2b, Supplementary Fig. S2). Therefore, the CD spectra of A-Z junctions is population-weighted depending on the amount of Z-conformation adopted by a particular RNA.

We also carried out CD experiments with a Zα construct where Tyr177 was mutated to alanine (Zα_Tyr177Ala_)—resulting in impaired Z-RNA binding capabilities^37,42^—which led to smaller absolute magnitudes of the shifts at these three wavelengths for the (CpG)_3_ RNA and resulted in a growth of the molar ellipticity at 266 nm for the A-Z junction (Fig. 2c). This growth likely occurred because Zα_Tyr177Ala_ can still bind to RNA, without causing the conformation switch to the Z-conformation (discussed later and shown by NMR in Supplementary Fig. S9).

A change in the CD spectra similar to that for the control A-Z junction was observed for AluSx1Jo and h43 as the Zα concentration was increased, indicative of the adoption of A-Z junctions in these RNAs (Fig. 2b). Measuring CD on stem-loops as in h43 also resulted in a decreased magnitude compared to measurement with only stems, because the loop restricts the conformationally accessible space. We verified this effect by comparing the CD curves for the AluSx1Jo duplex (Fig. 2b) and an engineered stem-loop version (Supplementary Fig. 3). Z-RNA adoption by h43 and AluSx1Jo was further supported by our negative control using Zα_Tyr177Ala_, which showed again an increase in the molar ellipticity at 266 nm and an entirely different behavior over the course of the titration (Fig. 2c and Supplemental Fig. S2).

To parametrize the extent of Z-RNA formation in AluSx1Jo and h43, we derived a Z-conformation score from the CD spectra. Specifically, we calculated the extent of Z-RNA formation (‘*E*_*Z*_’) as the growth in molar ellipticity at 285 and 295 nm and decrease in molar ellipticity at 266 nm of the fully saturated RNA, normalized to the molar ellipticity at 266 nm of the free form (calculated using Eq. 1 in “Methods” section). When the RNA is fully converted to a Z-conformation, as with (CpG)_3_ and (CpG)_6_, *E*_*Z*_ ~ 1 (Fig. 2d). Conversely, if the RNA remains in the A-form, *E*_*Z*_ ~ 0 (for a ((ApU)_6_, G_10_C_10_ duplex, as well as for cUUCGg and cGAAAg hairpins; Fig. 2d). A-Z 1 and A-Z 2 had values of 0.2 and 0.19, respectively, again, reflecting the fact that these RNAs do not fully adopt the Z-conformation (Fig. 2d). For AluSx1Jo and h43, *E*_*Z*_ ~ 0.3 and ~0.2, respectively, confirming further that these RNAs are very similar to the control A-Z junctions (Fig. 2d). The smaller changes in the CD spectra detected when using Zα_Tyr177Ala_ instead of Zα (Fig. 2b, c) resulted in a decrease in the *E*_*Z*_ score that was threefold to fourfold larger for AluSx1Jo, h43, A-Z 1, and A-Z 2 than for the (CpG)_3_ control (Fig. 2e, calculating using Eq. 2 in “Methods” section). The *E*_*Z*_ score therefore represents a suitable standard for evaluating the extent of Z-RNA formation for any sequence.

### Zα saturates the double-stranded region of AluSx1Jo, h43, and other ribosomal hairpins

We tested additional RNA fragments for their propensity to locally adopt a Z-RNA conformation upon addition of Zα. Using CD, we characterized the binding of Zα to chemically synthesized fragments corresponding to helices h41, h30, H25, and H66 (large subunit, LSU) from *E. coli* ribosomes, and h41, h30, and H66 from *H. sapiens* ribosomes, which had all been shown to bind to Zα in ribosome pull-down experiments^37^. We calculated the *E*_*Z*_ score of each tested fragment (Supplementary Figs. 3 and 4a; Supplementary Table 1).

**Fig. 3:**
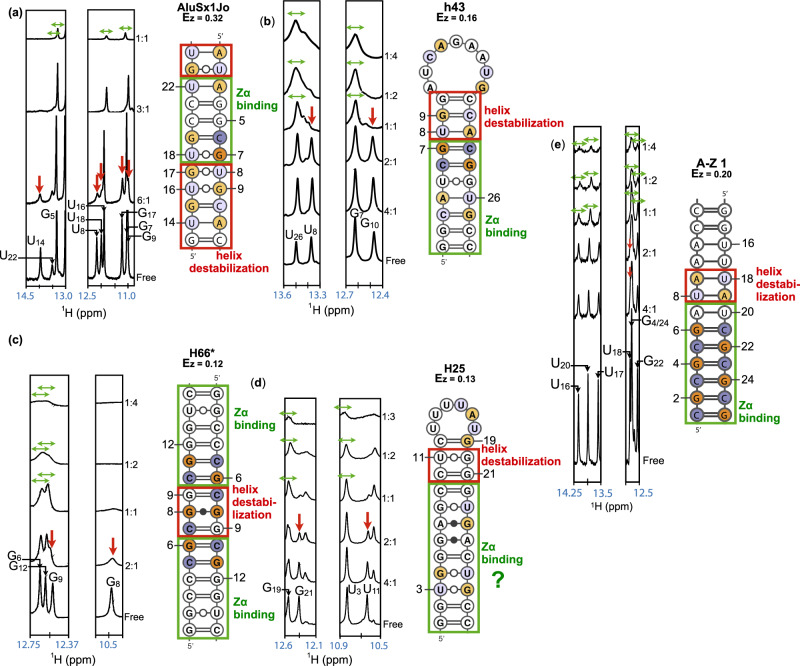
Pinpointing Zα binding sites and helix destabilization within various RNA sequence contexts. ^1^H-1D spectra of the following fragments: **a** AluSx1Jo, **b** h43 from *E. coli*, **c** H66* (extended duplex) from *H. sapiens*, **d** H25 from *H. sapiens*, **e** A-Z 1, at decreasing RNA:Zα ratios (right-hand side of the spectra slices). Peak disappearance (vertical red arrows) at low concentrations of Zα (below 1:1) caused by destabilization of the A-form helix required for A-Z junction formation is shown. Further line broadening at higher stoichiometric ratios of Zα to RNA (1:1, 1:2, 1:3, 1:4 RNA:Zα) indicate further binding of Zα proteins and growing complex size (green horizontal arrows). Full imino spectra are shown in Supplementary Fig. 8. For H25, due to the lack of information on the imino protons of G_6_ and G_24_, in addition to being unable to determine whether G4 is destabilized or coalesces with G28, the exact Zα binding region cannot be confirmed. NMR measurements were performed once as customary, and showed consistency with CD, ITC, and AUC experiments.

For these RNAs, the fully saturated spectra more closely resembled A-Z 1 and A-Z 2 than they did (CpG)_3_. *E*_*Z*_ scores ranged from ~0.1 to 0.47 (Supplementary Table 1), indicating that all tested fragments adopted A-Z junctions, but to various extents. Most fragments had *E*_*Z*_ values in the 0.1–0.2 range, unless they formed extended duplexes at the high concentrations required by the CD measurements, as was the case for H66 from *H. sapiens* (0.3) and for h41 from *E. coli* (0.47), which we monitored by AUC (see next paragraph). These duplexes were nonetheless interesting, as their predicted secondary structure (Supplementary Fig. 4a) suggested that in addition to recognizing YpR steps—of which these fragments had a high concentration—Zα may favor regions rich in non-canonical base pairs. In support of that possibility, the *E*_*z*_ score correlated well to the number of predicted non-Watson–Crick pairs (*R* = 0.78) and to RNA length (*R* = 0.75) (Supplementary Fig. 4b).

To determine the stoichiometric ratio of RNA and Zα within a complex, we conducted AUC experiments with Zα:RNA at a ratio of 6:1. We tested the following fragments: (CpG)_3_, cUUCGg hairpin, h43 (*E. coli*), H25 (*E. coli*), H66 (*H. sapiens*), H66 (*E. coli*), h41 (*H. sapiens* and *E. coli*), and the AluSx1Jo RNA. Specifically, we compared the fitted molecular weights from the AUC measurements with the theoretical molecular weights from increasing stoichiometric ratios of Zα:RNA. For each fragment except the negative cUUCGg control (major peak (78%) at 7.8 kDa, corresponding to unbound Zα), all potential Zα binding sites according to RNA length were occupied (Supplementary Fig. 5). For example, h43 bound to four Zα molecules (Supplementary Fig. 5), the maximum theoretically possible as its stem comprises 10 base pairs and each Zα spans ~4–5 base pairs^25,31^. However, while 96% of the complexes with h43 had a 4:1 ratio of Zα:RNA, the percentage of the saturated complex varied for the other fragments within 54–87% (Supplementary Fig. 5). In agreement with the CD data, this varying saturation range further suggests variable A-Z junction adoption within these RNAs.

We also measured h43 at 1:2 and 1:4 stoichiometric ratios of RNA:Zα, which gave molecular weights of 21.3 kDa (at 2:1 [RNA]:[Zα]) and 27.9 kDa (at 4:1 [RNA]:[Zα]), indicating 1:2, and 1:3 h43:Zα complexes, respectively (Supplemental Fig. S6). This suggests that while there are four binding sites for Zα on h43, the second pair of sites are less favorable than the first, as at a 1:4 stoichiometric ratio of h43:Zα, there is a 1:3 complex and the 1:4 complex only becomes stable by adding additional Zα proteins (at a 1:6 molar ratio of h43:Zα). This result highlights that while Zα is capable of binding a wide variety of RNA sequences, it still retains a sequence specificity that is dependent upon the ability of a particular sequence to adopt the Z-conformation.

### A-Z junction adoption in AluSx1Jo, H66, h43, and H25 involves destabilization of adjacent A-RNA

The observations that Zα binds to a variety of Alu and ribosomal sequences and that binding is correlated to the number of non-canonical base pairs raise the question of its sequence specificity. To investigate the context for Z-RNA formation at the nucleotide level upon Zα binding, we used 1D ^1^H-NMR because signatures of Z-DNA/RNA formation determined from previous work on (CpG)_n_ RNA/DNA repeats^23,43^ and B-Z junctions^31,32^ can be used as a reference for A-Z junction formation. Specifically, it was shown that Z-RNA formation within the (CpG)_3_ repeat mostly follows a two-step process. First, one Zα binds to one side of the repeat, which begins to convert the RNA to the Z-form. This event promotes binding of a second Zα molecule, which stabilizes the Z-conformation^23,43–45^. The conversion from the A-form to the Z-form in (CpG)_3_ DNA/RNA causes the imino proton of the guanine base within CpG repeats contacted by Zα to experience slow exchange between the chemical shift positions of the A- and Z-forms^23,43^. For B-Z DNA junctions, on the other hand, the adoption of the Z-DNA in the Z-forming sequence is inhibited by neighboring B-DNA^31,32^. Therefore, these regions must be destabilized by initial Zα binding before the DNA can adopt the Z-conformation, which causes the imino protons in such regions to disappear into the noise^31,32^. Following this initial destabilization, the Z-forming sequence is shifted to the Z-conformation and can either resemble the (CpG)_3_ case with slow exchange of the syn purines from the B-Z junction, or chemical shift perturbations (CSPs) can be observed instead, depending on the sequence^31,32^.

We followed Z-RNA formation within AluSx1Jo, h43, H66, H25, and A-Z 1 with increasing amounts of Zα (imino region of spectra in Fig. 3; 2D ^1^H-^1^H NOESY assignments and full 1D imino titrations in Supplementary Figs. S7 and S8). Following the addition of Zα up to a 1:1 ratio, the imino proton resonances of U_14_, U_22_, U_8_, U_18_, G_17_, and G_9_ of AluSx1Jo disappeared into the noise in a manner similar to what had been observed for the neighboring B-DNA in B-Z junctions^31^ and the A-Z 1 control, indicating those base pairs were destabilized (Fig. 3a, e). In addition, the imino protons in the Z-forming regions began to shift towards their bound positions concurrently with the disappearance of the imino protons in the destabilized regions, suggesting that the Z-formation and the destabilization steps were directly coupled. We observed the same behavior for the other three RNA constructs tested (Fig. 3b–d). We confirmed both effects were dependent upon Z-RNA formation as the reduction in the intensity of the same imino peaks when using Zα_Tyr177Ala_ was stunted relative to the wild-type, and the peaks never fully disappeared into the noise (Supplementary Fig. 9). In addition, the CSPs went in different directions than with wild-type Zα (Supplementary Fig. 9).

At higher stoichiometric ratios of RNA:Zα (1:1, 1:2, 1:3, 1:4), we also observed CSPs and line broadening (indicating the increase in complex size) in the base pairs that were not destabilized below 1:1 RNA:Zα. These results indicated loading of additional Zα proteins onto the different RNAs, in agreement with our CD and AUC results (Fig. 2 and Supplementary Figs. S2–S5). Plotting of the CSPs onto the secondary structures of the tested RNAs allowed us to determine the Zα binding sites (Fig. 3, green boxes). Similarly to what was observed for B-Z DNA junctions^31^ and A-Z 1, Zα binds to specific regions within AluSx1Jo, H66 (extended duplex), and h43, usually having a high concentration of G–C base pairs and one or more (YpR) steps. Binding also coincides with destabilization of the adjacent base pairs (Fig. 3). This is consistent with the formation of A-Z junctions within these RNAs and is supported by our CD results, which showed only partial Z-formation.

For H25, Zα appeared to target the lower portion of the stem which contained only two (YpR) steps and two G.A base pairs (Fig. 3d). However, the exact binding site could not be pinpointed as for the other fragments due to a lack of observable imino proton resonances for the G.A pairs at the temperature used for the titration. In addition, we cannot confirm whether the imino peak of G4 disappears into the noise due to Zα-dependent destabilization or whether it simply coalesces with G28 (Supplemental Fig. S8). The observation that the base-paired U27 peak does not disappear supports the latter conclusion, suggesting that the binding site comprises nine base pairs (four Zα binding sites) and would be in agreement with our AUC data (Supplementary Fig. S5). We did not observe any slow exchange between the A- and Z-form of any imino peaks in our RNA constructs as was the case for the (CpG)_3_ RNA^23^, with the exception of U11 and G21 of H25 (although we could not confirm that these were due to Z-RNA formation). This could be for a number of reasons, the most likely being that the formation of Z-RNA in RNAs with some sequence contexts is more dynamic and thus the conformations are exchanging on a faster timescale.

While the observed disappearance of imino peaks could be caused by line broadening due to intermediate exchange, the striking similarity to titrations done on the B-Z DNA junction^31^ suggests that this is not the case. In addition, we did not observe the re-emergence of these peaks at higher concentrations of Zα which we would expect as the complex becomes more stable in the case of intermediate exchange.

### Zα prefers dsRNA sequences adjacent to less stable helical elements

We noticed the regions being destabilized upon Zα binding usually occurred in regions with many non-canonical base pairs or near a helix end or loop (Fig. 3). Our CD results had revealed that the capping of a helix with a stable cUUCGg tetraloop decreased its *E*_*Z*_ score (Supplementary Fig. 3, Supplementary Table 1), and that *E*_*Z*_ correlated with the number of non-Watson–Crick pairs (Supplementary Fig. 4b). Together, these observations made us inquire whether Zα might be targeting stable dsRNA regions which are adjacent to regions with lower helical stability, as the thermodynamic barrier for Z-RNA adoption may be lower in such contexts.

**Fig. 4:**
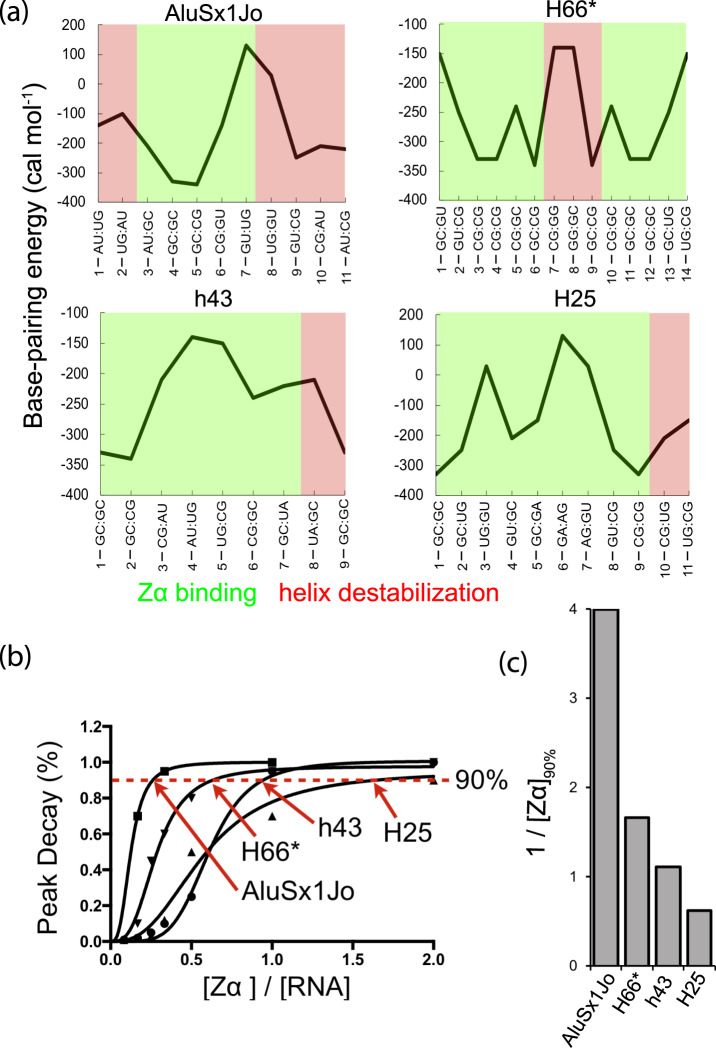
Destabilization of RNA helices by Zα correlates to base-pair stability. **a** Regions identified as either being bound (green) or destabilized (red) by Zα (referring to Fig. 3) are overlayed onto the predicted base-pair free energy (RNAeval^46^). **b** Imino peak height for proton signals in AluSx1Jo, H66* (extended duplex), h43, and H25 RNAs which are destabilized due to Zα-binding are shown versus Zα concentration. **c** The inverse of the Zα concentration at which 90% of the imino signals disappear into the noise is shown. Peak heights in **b** are from 1D NMR titrations (one measurement for each RNA).

Comparing the regions on AluSx1Jo and H66 which are bound or destabilized by Zα to predicted base-pair stabilities^46^ showed that Zα binds to the region(s) with generally the highest stability (low free energy) and tends to destabilize those with the lowest stabillity (Fig. 4a). The region of h43 which is destabilized by Zα-binding is immediately before h43’s nine-nucleotide loop (Fig. 4a), which lends further support to this hypothesis. H25 is an interesting case because the region that Zα appears to bind is relatively unstable (Fig. 4a). However, unlike AluSx1Jo, H66, and h43, H25 lacks a stretch of four or more Watson–Crick pairs and contains very few (YpR) steps. H25 may therefore be a poor Z-adopting RNA according to our NMR measurements, an observation that could not be fully resolved with the resolution of CD (H25 and H66 both have an *E*_*Z*_ score of ~0.1).

Tracking the disappearance of the imino peaks versus Zα concentration revealed that AluSx1Jo, h43, H66, and H25 are destabilized at different concentrations. Specifically, the concentration of Zα required to reduce the imino peak signals to 90% of their initial value is about 2.5-fold less for AluSx1Jo than for h43, twofold less than for H66, and eightfold less than for H25 (Fig. 4b). The concentration of Zα at which the A-form regions are destabilized in an RNA is likely directly related to the thermodynamic barrier of converting an RNA to the Z-conformation, as the neighboring A-form regions can interfere with the helical movement of the Z-adopting sequence, as was shown for B-Z DNA junctions^32^. Thus, the AluSx1Jo RNA is the best Z-adopting sequence from our selection, followed by H66, h43, and finally H25.

### Zα binds cooperatively to AluSx1Jo and h43 in the ~500 nM–2 µM range

**Fig. 5:**
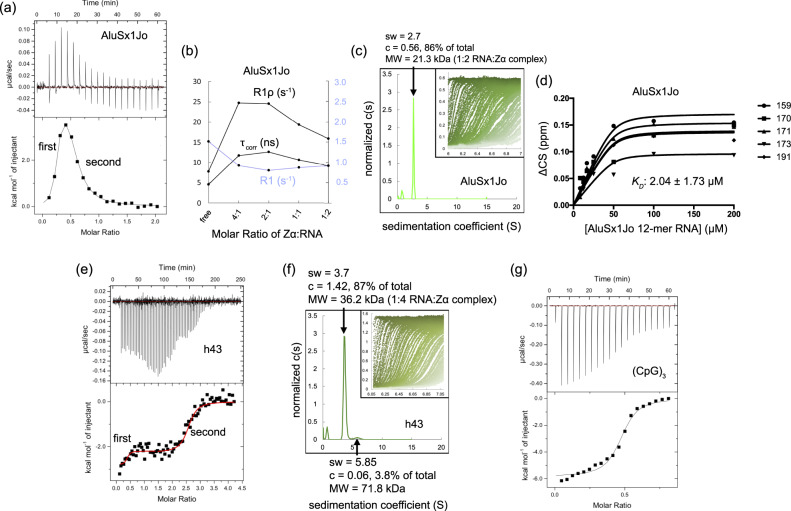
Zα binds to AluSx1Jo and h43 in the nanomolar to low-micromolar range. **a** ITC thermogram of AluSx1Jo titrated into Zα and fit to a two-site binding model. **b** Average (of the corresponding residue-specific values) longitudinal (*R*_1_), and rotating-frame ^15^N NMR relaxation rates (*R*_1ρ_), and the extracted effective overall correlation times (τ_corr_) are plotted versus the molar ratio of Zα:AluSx1Jo in the experiment (residue-specific *R*_1_, *R*_1ρ_, and τ_corr_ are shown in Supplementary Fig. 9). Measured values are from one set of relaxation rate experiments. **c** Sedimentation coefficient distribution as obtained from analytical ultracentrifugation (AUC) with a 1:6 molar ratio of AluSz1Jo:Zα. The inset shows the raw data from the AUC run with the window position on the *x*-axis and absorbance on the *y*-axis, and individual scans over time going from left to right. **d** Global *K*_d_ fit of chemical shift perturbations (CSPs) of binding-site residues from the ^15^N-HSQC titration of AluSx1Jo into Zα, assuming a two-site binding model. Values are determined from one ^15^N-HSQC titration. **e** Isothermal calorimetry (ITC) indicating the multiple Zα domain binding events for *E. coli* h43. **f** Sedimentation coefficient distribution as obtained from analytical ultracentrifugation (AUC) with a 1:6 molar ratio of h43:Zα. **g** ITC thermogram and fit from titrating the (CpG)_3_ RNA into Zα. All ITC parameters are given in Table 1. ITC thermograms are representatives of two measurements and AUC data for AluSx1Jo and h43 are determined from one measurement each.

Having determined that Zα induces novel A-Z junctions in our RNA constructs, we sought to characterize how Zα interacts with such elements. To characterize the binding of Zα to AluSx1Jo and h43, we used ITC and NMR measurements. For AluSx1Jo titration into Zα, these experiments showed an initial endothermic binding event up to a stoichiometric ratio of 2:1 of Zα:AluSx1Jo, followed by a second exothermic event which continued until saturation (Fig. 5a). The first binding event likely reflected a combination between binding, destabilization of the neighboring A-form regions, and Z-RNA formation (an endothermic cooperative process) at the saturating levels of Zα early in the titration. The second event captured the reshuffling of the populations from the 2:1 to 1:1 Zα:AluSx1Jo complex as the RNA began to saturate Zα. This model was supported by NMR measurements of the tumbling times (rotational correlation times, τ_corr_, calculated from Eq. 3 and the ratio of R2/R1 as described in “Methods” section) of Zα with different AluSx1Jo concentrations. These data indicated that the complex size was maximum at 2:1 Zα:AluSx1Jo, with a value of 12.6 ns, and decreased to 10.6 ns at 1:1 and finally 9.2 ns at 1:2 Zα:AluSx1Jo (Fig. 5b, residue-specific relaxation rates shown in Supplementary Fig. 10). These tumbling times were consistent with a 2:1 Zα:AluSx1Jo complex being formed at a 4:1 and 2:1 protein:RNA molar ratio, an intermediate complex between 2:1 and 1:1 at a 1:1 molar ratio of protein:RNA and a 1:1 complex at 1:2 protein:RNA. These results demonstrated cooperative binding, in agreement with the observed line broadening of the RNA imino peaks upon increasing concentrations of Zα (Fig. 3). The maximum of two bound Zα proteins was confirmed by AUC carried out at a 1:6 molar ratio of AluSx1Jo:Zα which showed that the majority of the RNA (85%) was in a complex of molecular weight 21.3 kDa (Fig. 5c) compared to the theoretical complex size of 22.5 kDa.

The ITC profile was best fit to a two-site binding model with a *K*_d_ of 1.14 ± 8.85 µM when the ratio of Zα:AluSx1Jo is ≤2 and 37.6 ± 103.8 nM when ≥2 (Table 1), although the large fitting error and other ITC parameters attests to a more complex binding behavior. Upon globally fitting NMR CSPs (calculated using Eq. 6 from “Methods” section) for five binding-site residues (His159, Lys170, Glu171, Asn173, and Thr191) from a ^15^N-HSQC titration of Zα and the AluSx1Jo RNA to a two-site binding model (Fig. 5d, individual per-residue fits shown in Supplementary Fig. 11), we obtained a *K*_d_ of 2.04 ± 1.73 µM. Together, these two independent measures revealed that the affinity of Zα for the AluSx1Jo RNA is in the mid-nanomolar to low-micromolar range.

**Table 1:** Parameters extracted from isothermal titration calorimetry experiments.

Interaction (cell/syringe)	*N*	*K*_*d*_ (nM)	Δ*H* (kcal mol^−1^)	*T*Δ*S* (kcal mol^−1^)	Δ*G* (kcal mol^−1^)
Zα/(CpG)_3_	0.4 ± 0.0	241.5 ± 1300.0	−6.0 ± 0.1	3.0	−9.0
h43/Za	0.4 ± 0.1/2.5 ± 0.0	538.0 ± 314.0/512.0 ± 1720.0	−0.6 ± 0.2/−2.3 ± 0.1	7.9/6.3	−8.6/−8.6
Zα/AluSx1Jo	0.2 ± 0.0/0.3 ± 0.0	37.6 ± 103.8/1140.3 ± 8849.6	−0.1 ± 0.2/5.9 ± 0.6	10.0/14.0	−10.1/−8.1

ITC measurements of Zα titrated into h43 showed exothermic binding heat with injections up to a Zα:RNA molar ratio of 2:1, followed by decreasing binding heat up to a ratio of 4:1 (Fig. 5e). This suggested that two Zα molecules were required before h43 could convert to the Z-conformation, with up to four Zα domains bound in the final state, in agreement with the AUC data (Fig. 5f and Supplemental Fig. S6). Doing the reverse ITC experiment (injecting h43 into Zα) showed a similar profile of multiple exothermic binding events (Supplemental Fig. S12). The thermogram could be fit to a multisite models, from which *K*_d_ values were extrapolated as 538 ± 314 nM for the first site and 512 ± 1720 nM for the second. Together, our binding analysis revealed that Zα binds specifically and cooperatively to the AluSx1Jo and h43 *E.coli* RNAs with affinities similar to those of Zα binding to perfect (CpG)_n_ repeats (*K*_d_ of 241.5 ± 1300 nM for r(CpG)_3_, Fig. 5g, Table 1; and *K*_d_ of 9 nM for (CpG)_6_, according to bio-layer interferometry measurements in ref. ^23^), although with more complexity.

### Binding of Zα to AluSx1Jo resembles binding to (CpG)_3_ but also to a B-Z DNA junction

Previous NMR studies showed that Zα interacts with (CpG)_3_ RNA and DNA repeats differently than B-Z DNA junctions^31,32^. For (CpG)_n_ repeats, significant CSPs (>0.2 ppm) to the backbone residues of Zα were observed for β1–α2 and β2–loop–β3 regions of Zα^23,32,43^, which reflect the binding of two Zαs and conversion to the Z-conformation. For B-Z DNA junctions, Zα experiences less extreme CSPs (<0.15 ppm) and the disappearance of residues 173–177 due to intermediate exchange, which was proposed to reflect an “initial contact conformation” where Zα binds to the Z-adopting sequence and destabilizes the neighboring B-form regions^32^.

**Fig. 6:**
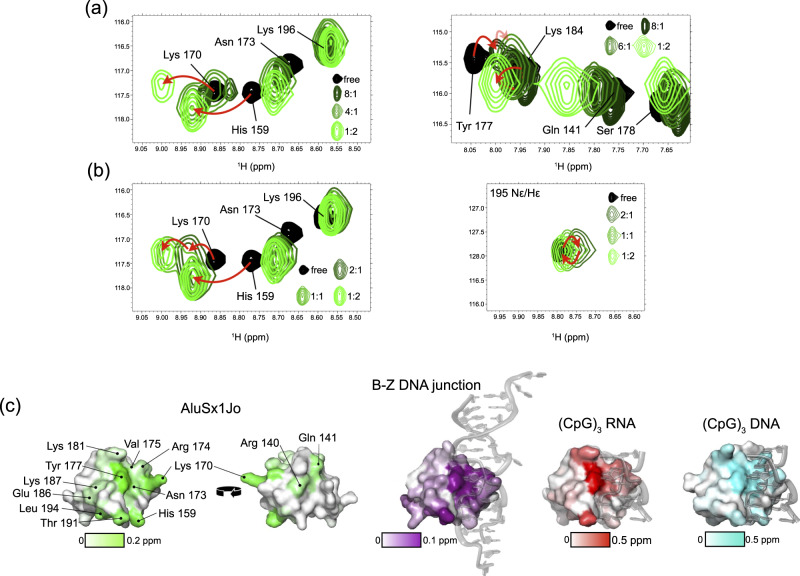
The binding of Zα to AluSx1Jo resembles both binding to the (CpG)_3_ RNA and a B-Z DNA junction. **a** Representative peaks and their perturbations from the Zα ^15^N-HSQC upon addition of AluSx1Jo. **b** Differences in peak positions between free Zα, 2:1, 1:1, and 1:2 Zα:AluSx1Jo. Red arrows help show the change in peak positions. **c** Residues undergoing chemical shift perturbation (CSP) and the CSP magnitudes (Supplementary Fig. 14a) from the ^15^N-HSQC titration are indicated on a surface plot of Zα (left, plotted on PDB: 2GXB^25^) and compared to CSPs from titrations of a B-Z DNA junction^32^ (second from left, PDB: 5ZUO^33^), the (CpG)_3_ RNA^23^ (second from right, PDB: 2GXB^25^), and the (CpG)_3_ DNA (23) (right, PDB: 3F21 (41)).

We employed NMR on ^15^N-labelled Zα to investigate how similar the binding site of AluSx1Jo on Zα is to that of (CpG)n repeats and to that of a B-Z DNA junction. The CSPs (calculated using Eq. 5 from “Methods” section) were non-linear and coupled with line broadening (Fig. 6a, full ^15^N-HSQC titration shown in Supplementary Fig. 13). These results indicated complex, multisite binding, consistent with our previous ITC, CD, AUC, and NMR results (Figs. 2–5). In addition, there were distinct differences in the peak positions between 2:1 and 1:1/1:2 Zα:AluSx1Jo for some of the binding interface residues (Fig. 6b), indicating differences in the backbone conformation of Zα between the state (one versus two Zα bound). This, in combination with our tumbling time analysis (Fig. 5b), suggests that at 2:1 Zα:RNA, two Zα molecules are bound to AluSx1Jo, which adopts a fully formed A-Z junction. At 1:1/1:2 Zα:RNA, however, Zα interacts with AluSx1Jo in the “initial contact conformation”, as was seen for B-Z DNA junctions^32^.

The largest CSPs measured from our ^15^N-HSQC titration of AluSx1Jo into Zα were mainly concentrated to the Z-RNA binding surface of Zα (amino acids 170–180, 190–194, green color in Fig. 6c, Supplementary Fig. 14). The affected amino acids were similar to those observed when bound to the (CpG)_3_ RNA^23^ (red, Fig. 6c) and the (CpG)_3_ DNA^23^ (cyan, Fig. 6c). However, their CSP magnitude was more similar to that observed for amino acids in the complex with a B-Z DNA junction^32^ (purple, Fig. 6c). In contrast to B-Z junctions (where residues 173–177 disappeared), only Tyr177 experiences intermediate exchange and line broadening beyond detection when bound to AluSx1Jo (Fig. 6a). This was confirmed by Chemical Exchange Saturation Transfer (CEST) measurements at 10:1 Zα:RNA, which showed that Tyr177 exhibited exchange on the micro–millisecond timescale with potentially multiple minor states, while Asn173, Arg174, Val175, and Ser178 showed little to no exchange (Supplementary Fig. 15). Hence, Zα interacts with AluSx1Jo in a manner that appears to have characteristics of both a B-Z DNA junction and the (CpG)_3_ RNA repeat.

Our analysis also revealed significant CSPs for Glu141 and His159. Perturbations in Glu141 were unexpected as the amino acid lies near the back side of Zα, adjacent to the binding helix. Glu141 may make specific interactions with A-Z junctions as this residue did not shift when bound to a B-Z DNA junction^32^. Notably, His159 had previously been shown to undergo chemical shift changes upon binding to (CpG)_3_ DNA but not RNA, possibly due to differences in the hydrogen bonding networks^23^. Our observation of a significant CSP for His159, combined with CSPs at residues 190–194 that do not occur when bound to d(CpG)_3_, suggests that Zα may invoke a hybrid binding mechanism between Z-DNA and Z-RNA.

### The binding of Zα to AluSx1Jo is more dynamic than for (CpG)_3_ RNA

**Fig. 7:**
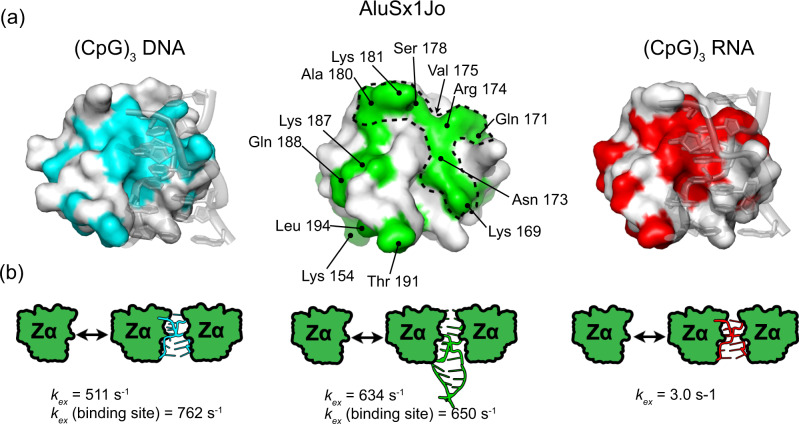
The binding of Zα to AluSx1Jo is more dynamic than to (CpG)_3_ RNA. **a** Residues for which CPMG data and exchange rates (*k*_ex_) were obtained between the free and the bound complex at 2:1 Zα:RNA are shown on surface plots of Zα bound to (CpG)_3_ DNA (left, PDB: 3F21^47^, *k*_ex_ rates from^23^), to (CpG)_3_ RNA (right, PDB: 2GXB^25^, *k*_*ex*_ rates from^23^), and for the AluSx1Jo RNA (middle, plotted on PDB: 2GXB^25^; residue-specific CPMG parameters for the AluSx1Jo are given in Supplementary Table 2 and fits are shown in Supplementary Fig. 16). **b** Globally fitted *k*_ex_ values for Zα binding to, from left to right, (CpG)_3_ DNA^23^ (all residues or only binding-site residues *k*_ex_ values are given), the AluSx1Jo RNA (globally fitted CPMG parameters are given in Supplementary Table 3), and (CpG)_3_ RNA^23^.

Because we did not observe slow exchange between the A-form and Z-form peaks in our RNAs (only CSPs and line broadening), we wondered whether the dynamics of Z-RNA formation might be faster in our constructs relative to the (CpG)_3_ RNA. To answer this question, we acquired Carr-Purcell-Meiboom-Gill (CPMG) measurements at 2:1 Zα:AluSx1Jo (two Zα residues symmetrically bound around a single Z-step) and compared them to previous CPMG measurements on the (CpG)_3_ DNA and RNA also measured at a 2:1 ratio of Zα:RNA^23^. CPMG profiles were globally fit (using Eq. 4 from “Methods” section) to give an exchange rate (*k*_ex_) between the bound and unbound states of 634 s^−1^ and major-state population of 96.2% (Fig. 7, Supplementary Fig. 16, Supplementary Table 2). Global fits from solely the binding-site helix (residues 169–180, 191) gave a *k*_ex_ and major-state population of 771 s^−1^ and 95.5%. These exchange rates were similar to what was measured for Zα binding to the (CpG)_3_ DNA (*k*_ex_ of 511 s^−1^ and *k*_ex_ for binding-site residues alone of 762 s^−1^), and were therefore roughly 200-fold higher than for the (CpG)_3_ RNA^23^. Thus, Z-RNA formation in our RNA constructs is more dynamic than for the canonical (CpG)_3_ repeat, and this may explain why we did not observe slow exchange between the A- and Z-form peaks in our imino titrations (Fig. 3).

## Discussion

**Fig. 8:**
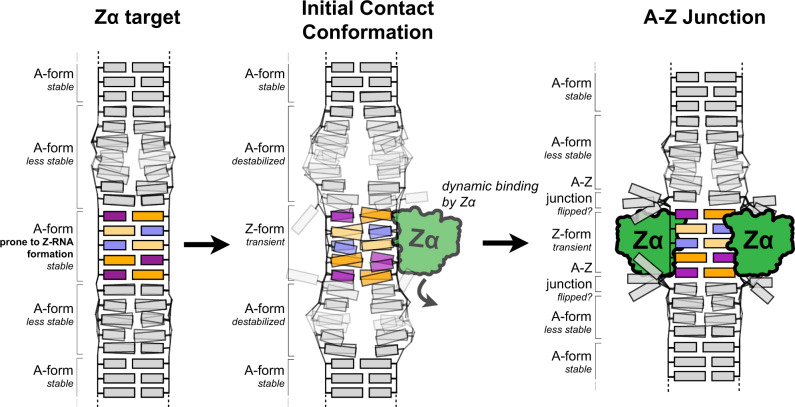
Model of the sequence specificity of Zα. Cartoon model depicting the proposed sequence specificity of Zα. A Z-RNA-prone region of dsRNA is flanked by regions of less stable A-form RNA (left). First, Zα dynamically interacts with the Z-prone sequence (usually comprising YpR steps and non-Watson–Crick pairs) in the initial contact conformation, which begins to convert the region to the Z-conformation, while also destabilizing the neighboring A-form regions (middle). This promotes the binding of another Zα molecule which then stabilizes the Z-conformation and converts the RNA into an A-Z junction (right), which may contain flipped out base pairs as has been seen for B-Z junctions^34^.

We have characterized various RNA fragments that bind to the Zα domain of ADAR1, even though they do not exclusively comprise CpG repeats. We have found that these RNAs likely adopt A-Z junctions, evidenced by the similarity of our imino titrations with Zα to those observed for B-Z DNA junctions^31,32^. NMR characterization of the binding of Zα to AluSx1Jo suggests that the mechanism for A-Z junction formation upon Zα binding lies somewhere between that of binding to the canonical (CpG)_3_ RNA and that of B-Z junction formation. In addition, our NMR results suggest that the initial interaction of Zα with such RNAs involves the proposed “initial contact formation”^31,32^ where Zα begins to destabilize the neighboring A-form regions, followed by the binding of an additional Zα molecule and adoption of the Z-conformation (Fig. 8).

Our findings suggest that a wider variety of sequence contexts than previously assumed are able to adopt a Z-conformation, as had been shown for Z-DNA^47^. This observation is in line with the adoption of Z-RNA-like steps by other sequences than CpG within a large variety of non-coding RNA^30^. This work also offers experimental evidence to support previous proposals that sequences, such as UpG adopt a Z-RNA conformation^36^. Whether the Z-RNA structures adopted by these diverse sequences are similar or represent various types of Z-conformations (as proposed from FRET measurements for CpG repeats^48^) will require structural determination of these complexes.

Even though the nucleotide sequences supporting A-Z junction arrangements are diverse, Z-RNA formation is still subject to a degree of specificity. We observed that Zα targets specific regions with dsRNA that are adjacent to regions which are predicted to be less thermodynamically stable (Figs. 3–4, 8). It has been shown that B-DNA next to a Z-forming sequence in B-Z junctions can present a significant thermodynamic barrier for Z-DNA formation^31,32^. It follows from our analysis that the stability of A-form regions adjacent to a Z-forming sequence in RNA would pose a similar barrier, and that Zα may favor binding to regions which can be converted to the Z-conformation more easily (dsRNA regions adjacent to regions with low stability). The sequence of the Z-adopting region is likely also important, as for Zα converting B- to Z-DNA^49^. The number of hydrogen bonds at the protein-DNA interface is maximal for CpG but minimal for TpA^29^. We similarly notice in our study that Alu adopts the Z-form at a lower protein concentration than H66 (extended duplex) > h43 > H25. From this analysis we conclude that (CpG)_n_ sequences are better Z-adopting sequences compared to other combinations, but that Gs in any configuration at the binding site (i.e., within UpG, ApG or GpG) tend to be preferred. Therefore, sequence specificity is likely driven by both the Z-forming propensity of the binding site and the stability of the adjacent regions (Fig. 8). We attempted to correlate the calculated *E*_*Z*_ scores to other factors in our tested RNA fragments, such as the number and identity of dinucleotide steps within the RNA. However, the only significant correlations to come out of this analysis were to RNA length and the number of non-canonical base pairs. This highlights the complexity in the factors which contribute to the ability of a particular RNA to adopt the Z-conformation, of which our simple analysis was unable to uncover.

This work supports the hypothesis that Z-RNA formation is a general feature of RNA. We know that Z-DNA conformations are readily adopted during replication and transcription due to unwinding of the two DNA strands^50^. So we could expect Z-RNA conformation to take place upon the many folding/unfolding events associated with the life of an RNA. Many crystal structures of non-coding RNAs reveal Z-RNA-like steps^30^, suggesting they participate in non-standard RNA structure and dynamic events. Our NMR data indicate that Z-RNA formation within the ribosomal and Alu stems is more dynamic than within duplexes made exclusively of CpG repeats. A dynamic binding of Zα could be a desirable feature for ADAR1, as being bound to only certain RNAs for an extended period of time would likely not be conducive to editing >10,000 sites within cellular RNAs.

If Z-RNA formation was a broad mechanism for subtle regulation of RNA expression and/or folding, it would certainly make sense that its sequence requirements would be somewhat loose. A loose RNA sequence requirement for Z-RNA formation would not be paradoxical to recognition by Zα if the sequence requirements for the recognition process were also loose. In fact, double-stranded RNA binding domains from ADAR2 and other proteins display somewhat relaxed sequence requirements^51^. Loose sequence requirements for Zα recognition may actually represent another illustration of the inherent “messy” nature of biology that would be at play during A-to-I editing^52^.

Widespread Z-RNA formation would influence the editing activity of ADAR1, which in turn would affect the proportion of edited versus unedited double-stranded RNA. This mechanism could participate in helping the cell to modulate the innate immune IFN response^4^. Our demonstration that Zα binds to non-CpG repeats at sites rich in non-Watson–Crick pairs and regions of lower stability is in agreement with earlier proposals that ADAR enzymes recognize their substrates in two separate events^39,40^. As such, the role of Zα could be to attract or deliver ADAR1 to a particular structure adjacent to sites that would thus eventually be recognized by the double-stranded RNA binding domains and the deaminase domain of ADAR1. Zα could increase the lifetime of ADAR1 on RNAs that already have been significantly edited, through binding to or near the mismatches produced by A-to-I editing.

Whether Zα binds to ribosomal stem-loops in vivo remains unclear. It is possible that results from the pull-down assays were biased towards ribosomal RNA sequences, as they are most abundant in RNA extracts^37^. On the other hand, Zα binding to ribosomal RNA could be relevant to ribosome assembly processes^37,53^, or to the fate of ribosomes in the stress granules, where ADAR1 and other Z-binding proteins are also located^54,55^. Studying Z-RNA formation in rRNA stem-loops helped us nonetheless with characterizing Zα binding to non-CpG repeat sequences and to identify similar regions in Alu elements.

Overall, pinpointing Z-RNA formation, occurrence, stability, and recognition by other macromolecules is crucial to getting the full picture of gene regulation. Once we better understand the structural underpinnings of Z-RNA formation, we will be able to determine how widespread such conformations are and what their role is within the cell.

## Methods

### Plasmid construction, protein purification, and experimental buffers

The N-terminal Zα domain of *Homo sapiens* ADAR1 in the pet-28a(+) plasmid (N-terminal 6x His-tag and thrombin cleavage site between His-tag and the Zα sequence) was a gift from Drs. Peter Dröge and Alekos Athanasiadis. Zα was expressed and purified similarly to^25,56^. Briefly, the plasmid was transformed and expresed in BL21(DE3) *E. coli.* The cultures were grown in Luria Broth and induced with 1 M IPTG at an OD_600_ of 0.6 and allowed to express Zα for 4 h at 37 °C, then centrifuged to collect the cell pellets. Pellets were resuspended in lysis buffer (50 mM Tris-HCl (pH 8.0), 300 mM NaCl, 10 mM Imidazole, 5 mM β-Mercaptoethanol (BME)) and sonicated. Lysate was centrifuged and the supernatant was applied to a His-trap column, washed with 40 mL of lysis buffer, 80 mL of wash buffer (50 mM Tris-HCl (pH 8.0), 1 M NaCl, 10 mM Imidazole, 5 mM BME), and eluted in 20 mL of elution buffer (50 mM Tris-HCl (pH 8.0), 300 mM NaCl, 500 mM Imidazole, 1 mM BME). The eluted fraction was then dialyzed into thrombin buffer (5 mM Tris-HCl (pH 8.0), 37.5 mM NaCl, 62.5 mM CaCl_2_, 0.5 mM DTT), and incubated with bovine thrombin (Millipore-Sigma, Burlington, MA) at room temperature overnight. The resulting samples were then reapplied to a His-trap column and the flow-through was collected. The flow-throughs were then concentrated to ~2 mL and applied to a Superdex 75 Gel Filtration Column (120 mL, GE Healthcare) and peak corresponding to pure Zα was collected and concentrated using an Amicon 3 kDa cutoff centrifugal filter (Millipore-Sigma, Burlington, MA). Zα_Tyr177Ala_ mutant was ordered from GenScript and prepared the same way as Zα. Proteins were dialyzed into 20 mM potassium phosphate (pH 6.4), 25 mM NaCl, 0.5 mM EDTA for NMR titrations (75 mM NaCl for Zα-Zβ, see below), and for ITC, proteins were dialyzed in the same beaker with the RNA (to match buffer conditions) in 20 mM potassium phosphate (pH 7.0), 25 mM NaCl, 0.5 mM EDTA, and 1 mM DTT. Proteins were concentrated using Amicon 3 kDa cutoff centrifugal filters. For CD and AUC, proteins were diluted into respective buffers from concentrated stocks. The buffers were as follows: 20 mM potassium phosphate (pH 6.4), 25 mM NaCl, 0.5 mM EDTA, 1 mM DTT for CD, and 20 mM potassium phosphate (pH 7.0), 25 mM NaCl, 0.5 mM EDTA, and 1 mM DTT for AUC.

### RNA preparation

RNA constructs were purchased from Horizon Discovery (Boulder, CO) with HPLC purification. RNAs for NMR titrations were dialyzed into 20 mM potassium phosphate (pH 6.4), 25 mM NaCl, 0.5 mM EDTA, and concentrated to 300 μL using Amicon 3 kDa cutoff centrifugal filters followed by heat denaturing at 95 °C for 1 min and rapid cooling on ice. D_2_O was added to 5% prior to NMR measurements. The RNAs for CD and AUC were resuspended at 1 mM in RNAse free ddH_2_O and diluted to the concentrations indicated for the experiments. For ITC, the RNAs were dialyzed in the same beaker as the proteins in order to match the buffer conditions and concentrated afterwards using 3 kDa cutoff centrifugal filters.

### Circular dichroism

All CD measurements were collected in 1-nm steps from 320 to 220 nm using a JASCO J-815 CD spectrometer (run using Spectra Manager version 2 (JASCO)) in a 0.1 cm quartz cuvette in 20 mM potassium phosphate (pH 6.4), 25 mM NaCl, 0.5 mM EDTA, and 1 mM DTT at 25 °C. Two scans were acquired and averaged. RNAs were heat denatured at 95 °C for 1 min followed by rapid cooling on ice and titration samples were prepared by incubating 50 µM of the RNA constructs with the specified amount of Zα or Zα_Tyr177Ala_ at 42 °C for 30 min and then bringing the samples down to 25 °C over a period of 20 min. Control experiments were run to ensure that the absorbance of Zα and Za_Tyr177Ala_ alone at the same concentrations was minimal in the 250–320 nm range (the range which reports on RNA secondary structure) (Supplementary Fig. 2). This indicated that changes in the CD spectra were due to conformational changes in the nucleic acid and not from the superposition of the RNA and Zα spectra. We tested our RNAs with 6 M sodium perchlorate which was shown to fully induce the Z-conformation in the (CpG)_n_ RNAs^57^.

We refer to either the RNA:Protein or the Protein:RNA ratio, with the first molecule mentioned being the one that is being titrated into, for CD, ITC, and NMR measurements.

### Extent of Z-formation calculation

To experimentally quantify the extent of Z-conformation present in an RNA construct, we introduced a Z-conformation score derived from CD spectra (*E*_*Z*_). *E*_*Z*_ is based on the CD intensities at wavelengths 285 and 295 nm (Int^285^ and Int^295^), both of which have been shown to be characteristic of Z-conformation^22,41^, and the decrease in intensity at 266 nm (Int^266^), which reports on the reduction of the A-form. We assumed that the growth of the intensities at 285 and 295 nm and decrease at 266 nm are proportional to the presence of Z-conformation with an offset accounting for intensities measured when no Z-conformation is present. We calibrated the following equation from CD intensities measured from (CpG)_3_, where the intensities reach plateaus at 100% Z-conformation:

1 $$E_Z \,=\, (1.800 \,\times\, {\mathrm{decay}}^{266} \,+\, 0.718 \,\times\, {\mathrm{growth}}^{285} \,+\, 1.109 \,\times\, {\mathrm{growth}}^{295})/3$$

where

$${\mathrm{decay}}^{266} \,=\, \left( {{\mathrm{Int}}_{{\mathrm{free}}}^{266}\,-\,{\mathrm{Int}}_{{\mathrm{bound}}}^{266}} \right)/{\mathrm{Int}}_{{\mathrm{free}}}^{266}$$

$${\mathrm{growth}}^{285} \,=\, \left( {{\mathrm{Int}}_{{\mathrm{bound}}}^{285}\,-\,{\mathrm{Int}}_{{\mathrm{free}}}^{285}} \right)/{\mathrm{Int}}_{{\mathrm{free}}}^{266}$$

$${\mathrm{growth}}^{295} \,=\, \left( {{\mathrm{Int}}_{{\mathrm{bound}}}^{295}\,-\,{\mathrm{Int}}_{{\mathrm{free}}}^{295}} \right)/{\mathrm{Int}}_{{\mathrm{free}}}^{266}$$

and the prefactors were chosen so that the *E*_*Z*_ score of the (CpG)_3_ RNA would be equal to one.

*E*_*Z*_ ideally takes values between 0 (no Z-conformation) and 1 (each nucleotide 100% in Z-conformation). Values between 0 and 1 indicate that the molecule is partially in Z-conformation. This may arise from different contributions from the various nucleotides such that a specific *E*_*z*_ score can be achieved by different Z patterns. For example, if only a subset of the nucleotides adopts Z-conformation at 100%, *E*_*Z*_ will be lower than 1.

An error of 0.1 was determined to be appropriate for the calculated *E*_*Z*_ scores by taking into account the difference between the (CpG)_3_ and (CpG)_6_ RNAs (which theoretically both have an *E*_*Z*_ score of 1) and the difference in the *E*_*Z*_ score between repeat measurements on h43 *E.coli*.

The fractional decrease in the *E*_*Z*_ score (Fig. 2e) was calculated by the following equation:

2 $${\mathrm{Fractional}}\,{\mathrm{decrease}}\,{\mathrm{in}}\,{\mathrm{the}}\,E_Z\,{\mathrm{score}} \,=\, \left( {E_{Z\,{\mathrm{Z}}\alpha {\mathrm{Tyr}}1{\mathrm{77Ala}}} \,-\, E_{Z\,{\mathrm{Z}}\alpha {\mathrm{WT}}}} \right)/E_{Z\,{\mathrm{Z}}\alpha {\mathrm{WT}}}$$

where *E*_*Z* ZαWT_ and *E*_*Z* ZαTyr177Ala_ are the *E*_*Z*_ scores determined from the CD measurements with the wild-type Zα and mutant Zα (Tyr177Ala), respectively.

Our method is based on the assumption that the RNA in question starts off predominantly in the A-form. For other conformations, our approximation would be less rigorous. We consider not only the changes in the molar ellipticities at 285 and 295 nm, but also at 266 nm because the loss of the A-form peak is directly related to the transition to the Z-conformation. For Z-RNA junctions where only a portion of the RNA adopts the Z-conformation, the growth at 285 and 295 is not as extensive compared to the (CpG)_n_ repeat (as was the case for A-Z 1 and A-Z 2, Fig. 2), making including the decrease at 266 nm more important. In addition, a change in the CD spectra, such as the stabilization of the A-form conformation can result in the peak increasing in height and broadening and consequently, increasing the molar ellipticity at 285 and 295 nm, thereby falsely contributing to the *E*_*Z*_ score. However, this also means that the *E*_*Z*_ score is sensitive to both the switch from the A- to the Z-conformation and also the destabilization of the neighboring A-RNA. While the *E*_*Z*_ score may be slightly underestimated by negative contribution to the molar ellipticity at ~290 nm at the higher concentrations of Zα (Supplementary Fig. S2), this contribution is relatively small and is mostly canceled out by the fact that we weigh the *E*_*Z*_ score according to the (CpG)_3_ control (which has the same concentration of Zα as the other constructs).

### Isothermal titration calorimetry

RNA constructs for ITC and the Zα protein were dialyzed overnight into 20 mM potassium phosphate (pH 7.0), 25 mM NaCl, 0.5 mM EDTA, and 1 mM DTT (in the same beaker to match buffers) and concentrated to ~500 µM using Amicon 3 kDa cutoff centrifugal filters. Binding heat was measured on a Malvern ITC200 instrument (run using ITC200 version 1.26.1 (Malvern)) at 25 °C and 750 RPM, with 180 s injection delays and a reference power of 10 µcals^−1^. The titrations of (CpG)_3_ into Zα were measured with twenty 2 µL injections of 200 µM RNA into 50 µM of protein. The titration of Zα into h43 *E. coli* was measured with eighty consecutive 0.5 µL injections of 1 mM Zα into 50 µM of RNA. The titration of h43 *E. coli* into Zα was measured with twenty 2 µL injections of 400 µM RNA into 20 µM of protein. The titration of AluSx1Jo into Zα was measured with twenty 2 µL injections of 200 µM RNA into 20 µM of protein. All ITC thermograms were analyzed and fit using Microcal Analysis version 7 SR4 (Origin); the details of fitting are detailed in ref. ^58^.

### Analytical ultracentrifugation

For the interactions between Zα and the different RNAs (Extended Data Fig. 5), the concentrations of Zα and the RNA tested were 2 and 12 μM, respectively, corresponding to a 1:6 ratio of RNA:protein, except for h43 which was measured at three different concentrations of Zα (4, 8, and 12 µM). Samples were loaded into a cell composed of standard 12 mm EPON centerpieces with quartz windows and sedimented at 50,000 RPM at 25 °C using an XL-I (Beckman Coulter) AUC instrument (run using ProteomeLab version 6.0 (Beckman)). UV absorbance was monitored at 260 nm for 16 h. Data were analyzed with SEDFIT (Version 14.7 g, NIH)^59^ using a specific volume which was normalized to the weight-average of RNA and protein^60^. The complex stoichiometry was chosen according to which theoretical weight was the closest to the measured weight. Error in the measured molecular weight by AUC can be caused by deviation of the predicted specific volume or viscosity of the sample from the actual values. In addition, if one of the binding sites is weaker than the others resulting in decreased site occupancy, this may cause a deviation in the observed molecular weight from the predicted complex size.

### NMR experiments

All NMR experiments were carried out on Varian 600 and 900 MHz spectrometers (run using VNMRJ version 4.2 Revision A (Agilent)) equipped with 5 mm triple resonance ^1^H/^13^C/^15^N cold probes with a Z-axis gradient as well as a Bruker 600 MHz spectrometer (run using TopSpin version 7 (Bruker))_equipped with a 5/3 mm triple resonance ^1^H/^13^C/^15^N/^19^F cryoprobe (CP2.1 TCI). 1D ^1^H NMR titrations for h43 *E. coli*, H66 *H. sapiens*, and H25 *E. coli* with Zα were carried out on the Varian 600 MHz spectrometer, while the titration for the AluSx1Jo RNA was done on the Bruker 600 MHz spectrometer using a W5 scheme for water suppression (RNA concentration was 500 μM for all). The number of scans for all titration points was 128 with a relaxation delay of 1.6 s, and the spectral width was 24 ppm. 2D ^1^H-^1^H NOESY spectra were recorded on the Varian 900 MHz spectrometer for h43 *E. coli*, the Varian 600 MHz spectrometer for H66 *H. sapiens* and H25 *E. coli*, and the Bruker 600 MHz spectrometer for the AluSx1Jo RNA (RNA concentration was 1 mM for all). The 2D ^1^H-^1^H NOESY recorded for h43 *E. coli* was acquired with a mixing time of 200 ms, 1470 × 800 complex points (399 of the points were actually collected following a 50% NUS sampling scheme generated using the Schedule Generator from the Wagner group: http://gwagner.med.harvard.edu/intranet/hmsIST/gensched_new.html), a 1.3 s recycle delay, and 32 scans. The spectral widths were 22 × 22 ppm for both ^1^H dimensions. The 2D ^1^H-^1^H NOESY recorded for H66 *H. sapiens* was acquired with a mixing time of 320 ms, 1386 × 400 complex points (162 of the points were collected following a 40% NUS sampling schedule), a 1.3 s recycle delay, and 32 scans. The spectral widths were 20 × 20 ppm for both ^1^H dimensions. The 2D ^1^H-^1^H NOESY recorded for H25 *E. coli* was acquired with a mixing time of 200 ms, 1396 × 400 complex points (162 of the points were collected following a 40% NUS sampling schedule), a 1.3 recycle delay, and 32 scans. The spectral widths were 20 × 20 ppm for both ^1^H dimensions. The 2D ^1^H-^1^H NOESY recorded for AluSx1Jo was acquired with a mixing time of 300 ms, 1024 × 400 complex points, a 2 s recycle delay, and 32 scans. The spectral widths were 24.5 × 24.5 ppm for both ^1^H dimensions. 1D spectra from the Bruker spectrometer were processed using TopSpin; all other data were processed using the NmrPipe/NmrDraw/NlinLS package version 10.9^61^. All NUS data were reconstructed using the hmsIST software^62^ (a part of NMRPipe). All assignments were done in ccpNmr analysis version 2.4.2^63^.

For the titration of AluSx1Jo into Zα, all NMR measurements were carried out on the Varian 900 MHz spectrometer. The ^15^N-HSQC spectra for the titration were measured with 1048 (^1^H) × 60 (^15^N) complex points with a 1.6 s recycle delay and 32 scans. The spectral widths were 16 × 35 ppm for the ^1^H and ^15^N dimensions, respectively. The concentration of Zα was 200 μM. The R_1_ relaxation experiments were measured with 1048 (^1^H) × 64 (^15^N) complex points, a recycle delay of 2 s, 16 scans, and relaxation delays of 0, 100, 200, 300, 400, 500, 600, 700, 800, 900, 1000, and 1200 ms. The spectral widths were 16 × 35 ppm for the ^1^H and ^15^N dimensions, respectively. The R_1ρ_ relaxation experiments were measured with 1048 (^1^H) × 64 (^15^N) complex points, a recycle delay of 2 s, 32 scans, and relaxation delays of 0, 10, 20, 30, 40, 60, 80, 100, 120, and 160 ms. The spectral widths were 16 × 35 ppm for the ^1^H and ^15^N dimensions, respectively. During the R_1ρ_ relaxation time, a ^15^N spin-lock field of 1500-Hz strength was applied. The transverse relaxation rate *R*_2_ was calculated from *R*_1_ and *R*_1ρ_ using the following equation:

3 $$R_2 \,=\, R_{1\rho } \,+\, \left( {R_{1\rho }\,-\,R_1} \right) \,\times\, {\mathrm{tan}}^2(\theta )$$

where θ = tan^−1^(2πΔv/γ_N_*B*_*1*_), Δv is the resonance offset, |γ_N_*B*_*1*_/2π| is the strength of the spin-lock field B_1_, and γ_N_ is the gyromagnetic ratio of the ^15^N spin.

*τ*_*corr*_ was calculated from the ratio of *R*_2_/*R*_1_^64^. The *τ*_*corr*_ for free Zα was calculated from *R*_1_/*R*_1ρ_ measurements done with a 500 μM Zα sample instead of the 200 μM sample used in the titration.

The CPMG experiment at 2:1 Zα:AluSx1Jo (1 mM Zα, 500 μM AluSx1Jo) was measured on the 600 MHz Bruker spectrometer with 1024 (^1^H) × 64 (^15^N) complex points, a recycle delay of 1.2 s, 32 scans, and 11 ν_CPMG_ values (*T* = 40 ms) ranging from 10 to 1000 Hz. The spectral widths were 16 × 35 ppm for the ^1^H and ^15^N dimensions, respectively. Dispersion profiles were fit to a two-state fast CPMG exchange model with the following equation:

4 $$R_{2{\mathrm{eff}}} \,=\, R_{2{\mathrm{a}}} \,+\, p_{\mathrm{a}} \,\times\, \left( {1 \,-\, p_{\mathrm{a}}} \right) \,\times\, \left( {\Delta \omega ^2} \right)/k_{{\mathrm{ex}}} \,\times\, \left\{ {1 \,-\, \left( {4 \,\times\, {\upnu}_{{\mathrm{CPMG}}}/k_{{\mathrm{ex}}}} \right) \,\times\, \left( {\tanh \left( {k_{{\mathrm{ex}}}/{\upnu}_{{\mathrm{CPMG}}}/4} \right)} \right)} \right\}$$

where *R*_2a_ and p_a_ are the *R*_2_ relaxation rate and the population of state A, Δω is the difference in the chemical shift between states A and B, *k*_*ex*_ is the rate of exchange between states A and B, and ν_CPMG_ is the effective field strength of the refocusing pulse train.

The ^15^N-CEST experiment at 10:1 Zα:AluSx1Jo (1 mM Zα, 100 μM AluSx1Jo) was measured on the 600 MHz Bruker spectrometer with 1024 ^(1^H) × 64 (^15^N) complex points, a 1 s recycle delay, and 16 scans. The spectral widths were 16 × 35 ppm for the ^1^H and ^15^N dimensions, respectively. A weak ^15^N *B*_1_ field of 5 Hz strength was applied during a 400 ms relaxation time. A total of 66 datasets were acquired corresponding to a chemical shift range of 102–132 ppm with steps of 0.5 ppm. From 115 to 116 ppm, the steps were decreased to 0.25 ppm to acquire additional points around Tyr177. In all experiments, ^1^H decoupling was achieved using a 90_x_240_y_90_x_ composite pulse.

### *K*_*D*_ fitting of NMR titration points

CSPs from the ^15^N-HSQC titration of AluSx1Jo into ^15^N-labelled Zα were calculated using the following equation^65,66^:

5 $${\mathrm{CSP}} \,=\, \sqrt {\left( {{\updelta }}_{{\mathrm{H}},{\mathrm{free}}} \,-\, {\updelta}_{{\mathrm{H}},{\mathrm{bound}}} \right)^{2} \,+\, 0.2\left( {{\updelta }}_{{\mathrm{N}},{\mathrm{free}}} \,-\, {\updelta}_{{\mathrm{N}},{\mathrm{bound}}} \right)^{2}}$$

where *δ*_*H*_ is the chemical shift of a peak in the ^1^H dimension, and *δ*_*N*_ is the chemical shift of a peak in the ^15^N dimension. The *K*_*D*_ was determined by fitting CSPs to the following equation^66^:

6 $${\mathrm{CSP}} \,=\, {\mathrm{CSP}}_{{\mathrm{max}}} \,\times\, \frac{{\left( {K_D \,+\, \left[ L \right] \,+\, \frac{{\left[ P \right]}}{2}} \right) \,-\, \sqrt {\left( {\frac{{[P]}}{2}} \right)^2 \,-\, 4 \,\times\, \left[ L \right] \,\times\, \frac{{[P]}}{2}} }}{{2 \,\times\, \frac{{[P]}}{2}}}$$

Where [*P*] is the concentration of protein in solution (divided by two because the ligand has two binding sites), [*L*] is the ligand concentration, and CSP_max_ is the maximum CSP measured over the series of titration points.

### Comparison of CSPs between AluSx1Jo titration and (CpG)_3_ DNA and RNA

For comparison of our measured CSPs from ^15^N-HSQC titrations of Zα with the AluSx1Jo RNA and those measured for the (CpG)_3_ DNA and RNA^23^ and the B-Z DNA junction^32^, CSPs were estimated from the published bar graphs showing CSPs vs residue number.

### Prediction of base-pair-specific free energy of folding for AluSx1Jo, h43, H66, and H25

The base-pair-specific stability energies for AluSx1Jo, h43, H66, and H25 (Fig. 4) were predicted within RNAeval using default parameters^46^ (last accessed at http://rna.tbi.univie.ac.at on September 10, 2020) and plotted vs. base-pair identity. The regions identified as being bound and destabilized by Zα binding were overlayed over the plots.

### Reporting summary

Further information on research design is available in the Nature Research Reporting Summary linked to this article.

## Supplementary information

Supplementary information

Peer Review File

Reporting Summary

## Data Availability

The data that support this study are available from the corresponding authors upon reasonable request. The datasets generated during and/or analysed during the current study are available in the Dryad repository, with the identifier 10.5061/dryad.pvmcvdnk4. The crystal structures used in Figs. 6 and 7 (2GXB, 5ZUO, 3F21) are available from the RCSB. Source data are provided with this paper.
