# Supplementary material for: Recognition of non-CpG repeats in Alu and ribosomal RNAs by the Z-RNA binding domain of ADAR1 induces A-Z junctions
Source: Nat Commun. 2021 Feb 4;12:793. doi: 10.1038/s41467-021-21039-0 (PMC7862695; doi:10.1038/s41467-021-21039-0)
Supplement: Supplementary file 1 — Supplementary information [file 41467_2021_21039_MOESM1_ESM.pdf]

Supplementary information for:

Recognition of non-CpG repeats in Alu and ribosomal RNAs by the Z-RNA binding domain of ADAR1 induces A-Z junctions

Parker J. Nichols, Shaun Bevers, Morkos Henen, Jeffrey S. Kieft, Quentin Vicens & Beat Vögeli

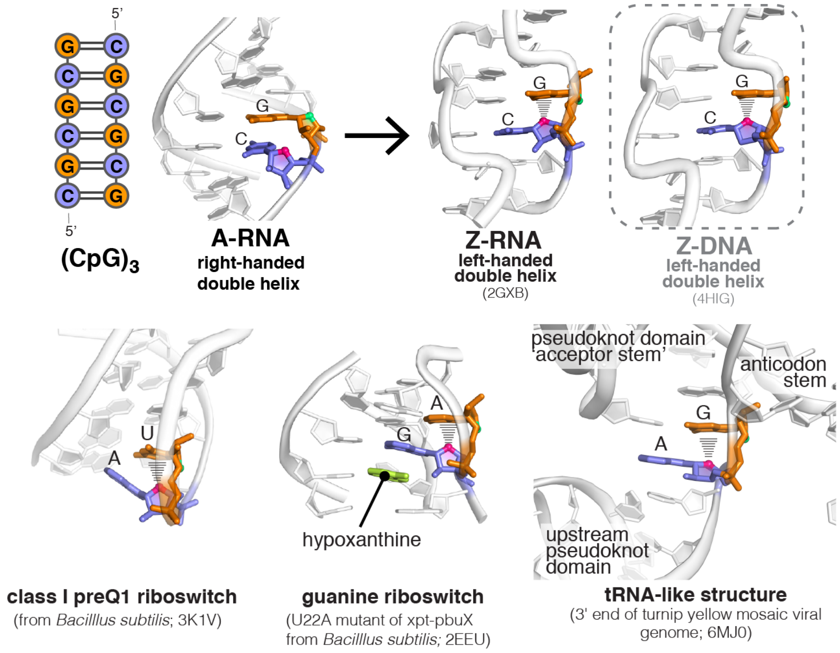

Supplementary Figure S1. Z-RNA conformations occur at the level of dinucleotides in various contexts. (Top) Comparison of A-RNA, Z-RNA and Z-DNA within double-helical contexts made of CpG repeats. (Bottom) Dinucleotides in Z-like conformations at critical locations within large RNAs. Position of the nucleotide at the 5' or 3' end of the dinucleotide shown in orange and blue, respectively (stacking interactions from the O4' atoms (in pink/green) shown as black lines). 4-letter PDB codes indicated.

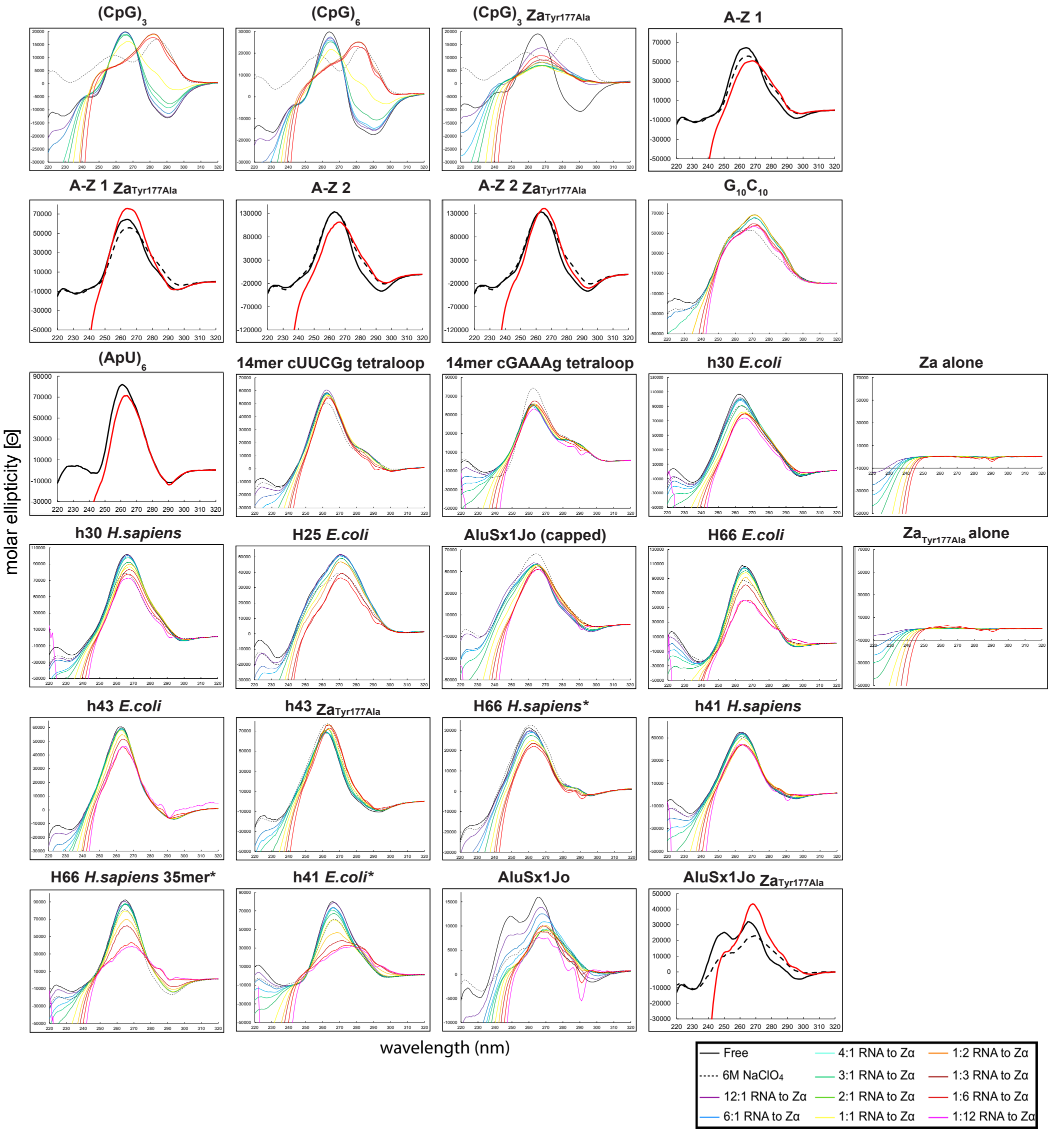

Supplementary Figure S2. Circular dichroism titrations of Zα. CD spectra at the various RNA:protein concentrations indicated for all of the RNAs studied. Unless otherwise specified, all titrations were carried out with Zα. Only the free RNA and 1:6 RNA:Zα points were collected for (ApU)$_6$. For A-Z 1 A-Z 2 with WT Zα, and AluSx1Jo with ZαTyr177Ala, only free RNA, 6M sodium perchlorate, and 1:6 RNA:Zα points were measured. * Indicates that the RNA forms a duplex instead of the expected stem-loop (determined by AUC, Supplementary Figures S4, S5). Measurements were repeated twice for (CpG)$_3$, (CpG)$_6$, and h43 *E.coli*.

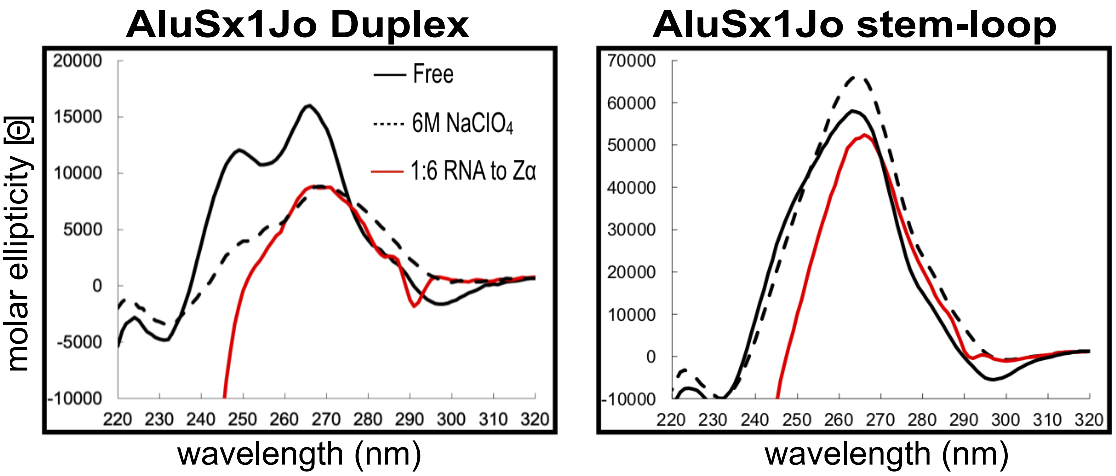

Supplementary Figure S3. CD spectra titrations comparing the AluSx1Jo Duplex and AluSx1Jo stem-loop RNAs. CD spectra of AluSx1Jo duplex (Figure 2) and its stem-loop version (Supplementary Figure S4c), either free or in the presence of 6M sodium perchlorate or 1:6 RNA:Zα.

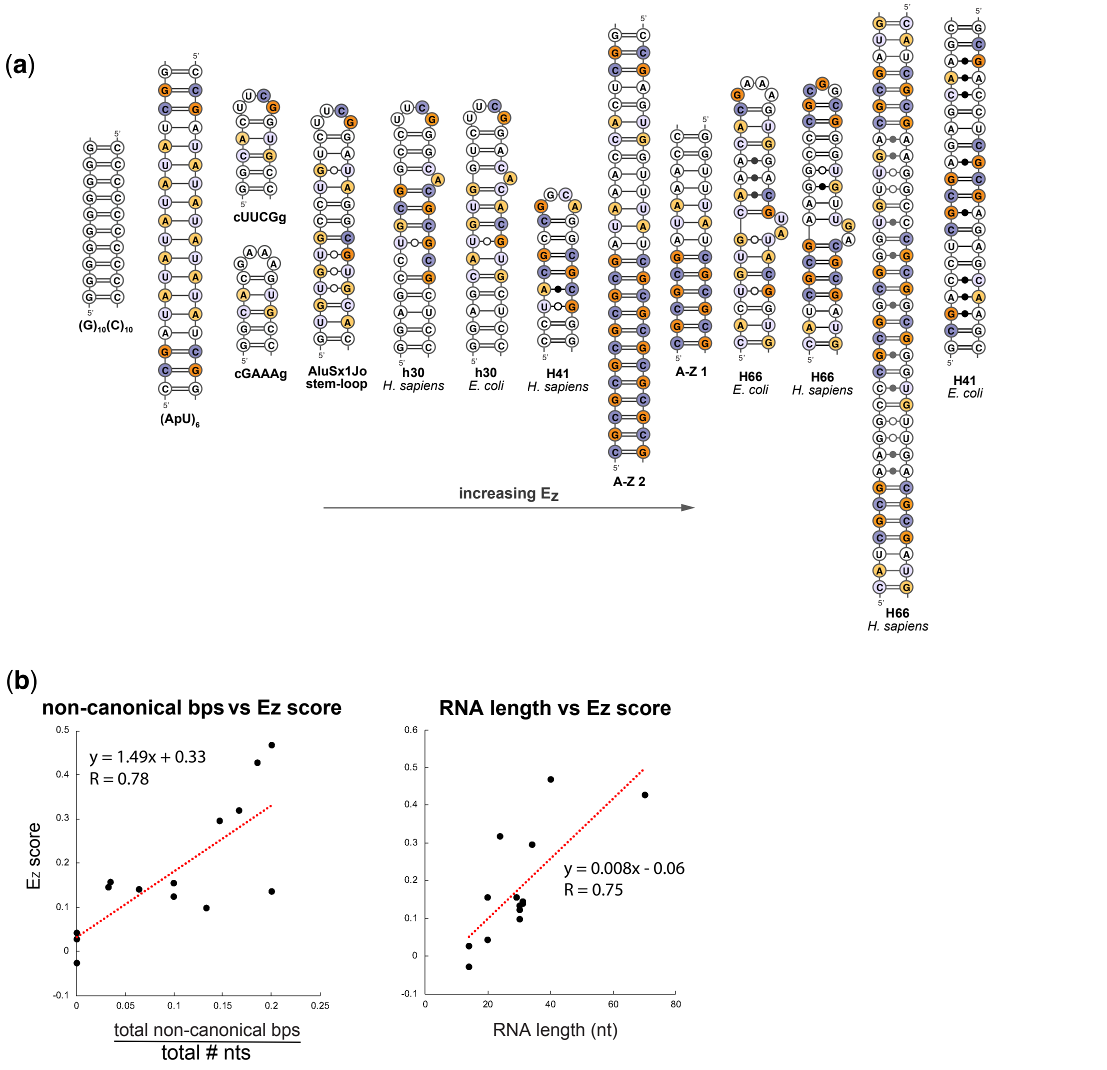

Supplementary Figure S4. Zα binds to double-stranded regions with a variety of sequences. (a) Secondary structures of a subset of the surveyed RNA fragments (complement shown in Figures 2 and 3) organized according to increasing E_Z score (Supplementary Table S1). (b) Linear correlation plots between the number of non-canonical base pairs or the total number of nucleotides and the E_Z score. Pearson's correlation coefficients were calculated in excel from E_Z scores determined from CD measurements of sample RNAs (one CD measurement per RNA).

Predicted RNA:Zα Complex Sizes

Constuct	free	1:1	1:2	1:3	1:4	1:5	1:6	1:8	1:10
**Zα**	7.3 kDa								
**(CpG)3 Duplex**	4.2 kDa	11.5 kDa	**18.8 kDa**	26.1 kDa	33.4 kDa				
**14mer cUUCGg tetraloop**	**4.7 kDa**	12 kDa	19.3 kDa	26.6 kDa	33.9 kDa				
**h43 *E.coli* 29mer Stemloop**	9.6 kDa	16.9 kDa	24.2 kDa	31.5 kDa	**38.8 kDa**	46.1 kDa	53.4 kDa		
**H25 *E.coli* 30mer Stemloop**	9.9 kDa	17.2 kDa	24.5 kDa	31.8 kDa	**39.1 kDa**	46.4 kDa	53.7 kDa		
**H66 *H.sapiens* 30mer Duplex**	10.0 kDa	17.3 kDa	24.6 kDa	31.9 kDa	**39.2 kDa**	46.5 kDa	53.8 kDa		
**H66 *H.sapiens* 35mer Stemloop**	11.6 kDa	18.9 kDa	26.2 kDa	33.5 kDa	40.8 kDa	48.1 kDa	55.4 kDa		
**H66 *H.sapiens* 70mer Duplex**	23.0 kDa	30.03 kDa	37.6 kDa	44.9 kDa	**52.2 kDa**	59.5 kDa	66.8 kDa	81.4 kDa	**96 kDa**
**H66 *E.coli* 34mer Stemloop**	11.2 kDa	18.5 kDa	25.8 kDa	33.1 kDa	40.4 kDa	**47.7 kDa**	55.0 kDa		
**H66 *E.coli* 68mer Duplex**	22.3 kDa	29.6 kDa	36.9 kDa	44.2 kDa	51.5 kDa	58.8 kDa	66.1 kDa		
**h41 *E.coli* 20mer Stemloop**	6.7 kDa	14.0 kDa	21.3 kDa	28.6 kDa	35.9 kDa	43.2 kDa	50.5 kDa		
**h41 *E.coli* 40mer Duplex**	13.2 kDa	20.5 kDa	27.8 kDa	35.1 kDa	42.4 kDa	**49.7 kDa**	57 kDa		
**h41 *H.sapiens* 20mer Stemloop**	6.7 kDa	14.0 kDa	21.3 kDa	28.6 kDa	35.9 kDa	43.2 kDa	50.5 kDa		
**h41 *H.sapiens* 40mer Duplex**	13.2 kDa	20.5 kDa	**27.8 kDa**	35.1 kDa	42.4 kDa	49.7 kDa	57 kDa		
**AluSx1Jo 12mer Duplex**	7.9 kDa	15.2 kDa	**22.5 kDa**	29.8 kDa	37.1 kDa				

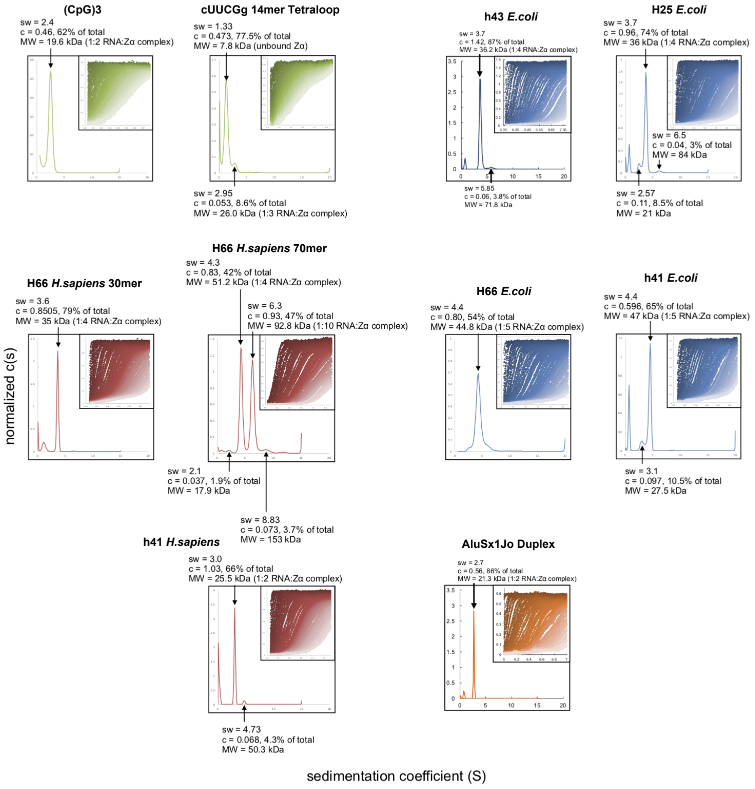

Supplementary Figure S5. Analytical ultracentrifugation of RNA:Zα saturated complexes. (Left) Predicted molecular weights for the RNA:Zα complexes measured. (Right) SEDFIT analysis of AUC runs for each RNA-Zα pair. AUC runs of each pair were analyzed and the peak containing the highest proportion of sample by area is highlighted on the table. Rows with no highlighted molecular weight are experiments where the molecular weight of the majority peak did not match any of the predicted molecular weights. The insets show the raw data from the AUC run with the window position on the *x*-axis and absorbance on the *y*-axis, and scans over time going from left to right. AUC measurements were measured once per RNA.

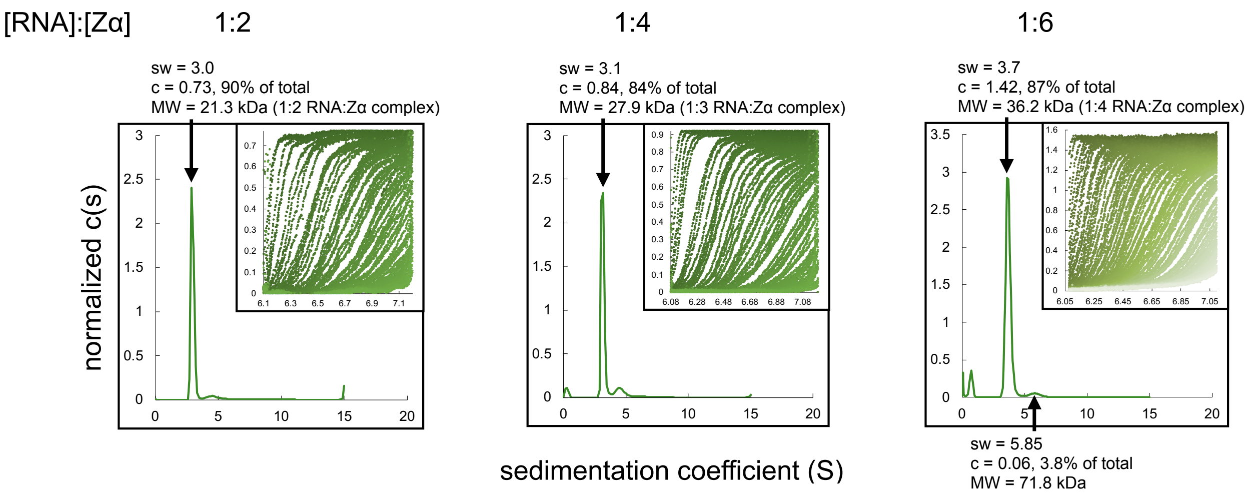

Supplementary Figure S6. Analytical ultracentrifugation of h43 at different RNA:Zα concentrations. SEDFIT analysis of AUC runs for h43 with 1:2, 1:4, and 1:6 ratios of [RNA]:[Zα]. The corresponding complex size (theoretical complex molecular weights are shown in Figure S5) is indicated next to the measured molecular weight. The insets show the raw data from the AUC run with the window position on the *x*-axis and absorbance on the *y*-axis, and scans over time going from left to right.

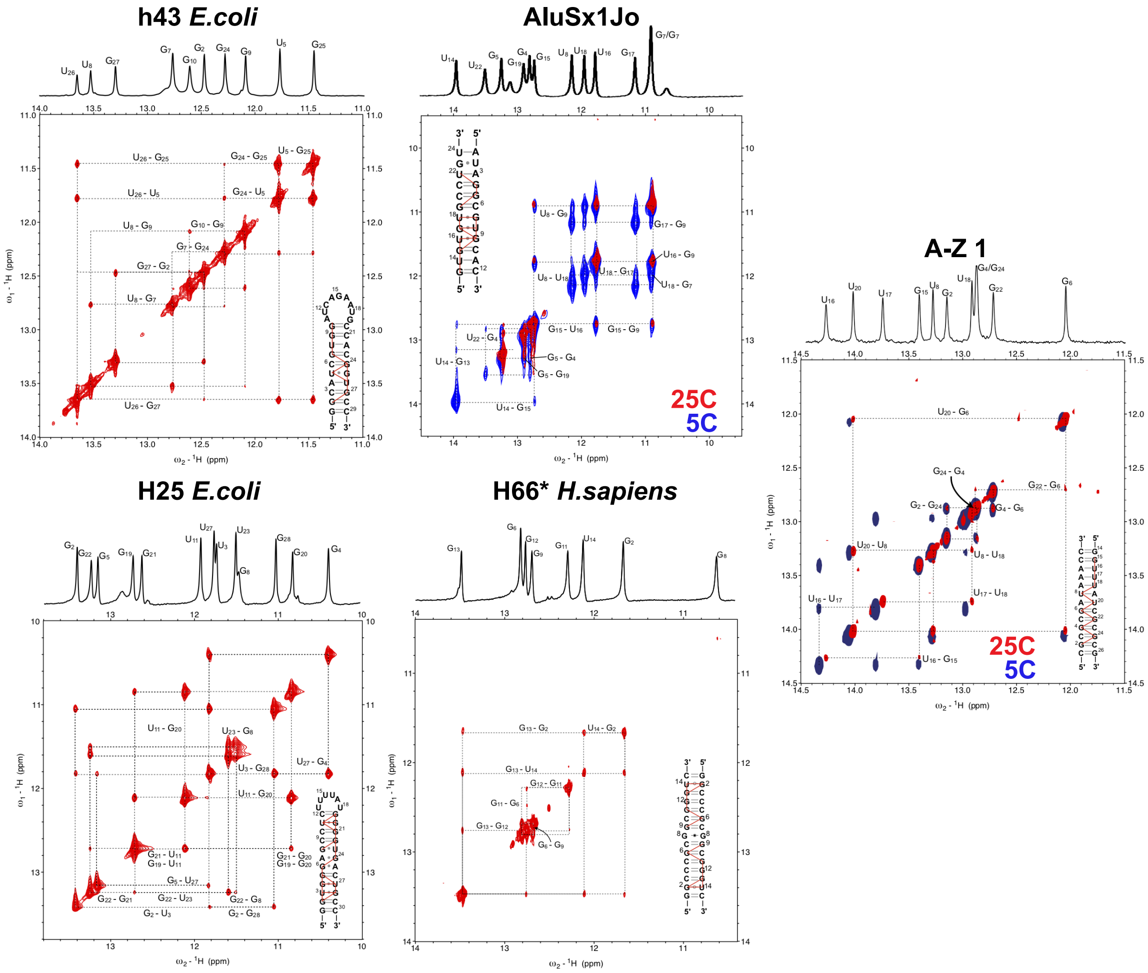

Supplementary Figure S7. 2D [^{1}H,^{1}H]-NOESY imino spectra and assignments. The imino regions of the NOESY spectra with mixing times of 300 ms for h43 *E. coli* (top, left), AluSx1Jo (top, right), H25 *E. coli* (bottom, left), H66* (extended duplex) *H. sapiens* (bottom, right), and A-Z 1 (middle right) are shown. Imino proton connectivities and assignments are shown with dashed lines and illustrated as red lines on the 2D secondary structures shown within each box. 1D spectra for each RNA with peak assignments are shown above the NOESY spectra.

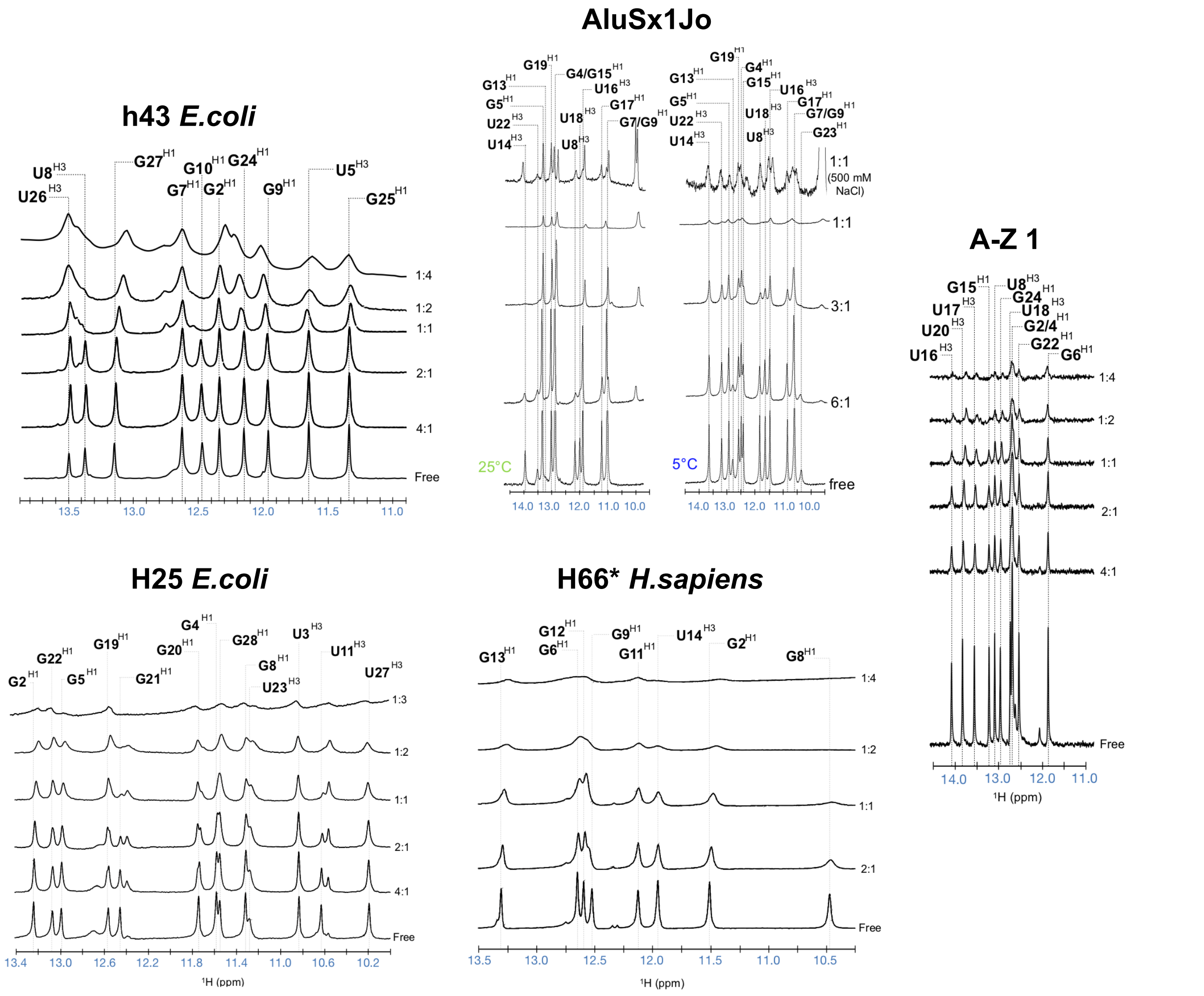

Supplementary Figure S8. Full 1D imino spectra from Zα titrations. The full imino regions of the 1D ^{1}H-NMR titrations are shown for h43 *E. coli*, AluSx1Jo, H25 *E. coli*, H66* (extended duplex) *H. sapiens*, and A-Z 1. Imino proton assignments are indicated with dashed lines. The ratio of RNA:Zα is indicated on the right-hand side of each trace.

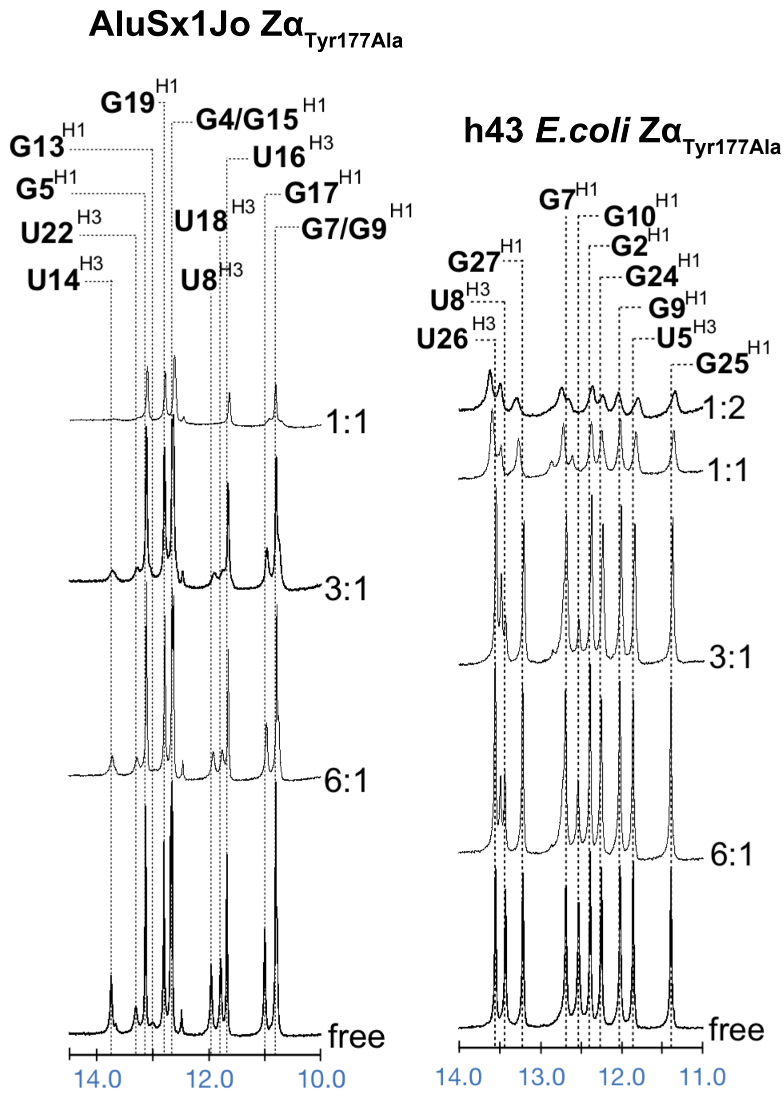

Supplementary Figure S9. 1D imino spectra of AluSx1Jo and h43 from titrations with $Z\alpha_{Tyr177Ala}$. The full imino regions of the 1D ^{1}H-NMR titrations are shown for h43 *E. coli*, AluSx1Jo with increasing concentrations of $Z\alpha_{Tyr177Ala}$. Imino proton assignments are indicated with dashed lines. The ratio of RNA:$Z\alpha_{Tyr177Ala}$ is indicated on the right-hand side of each trace.

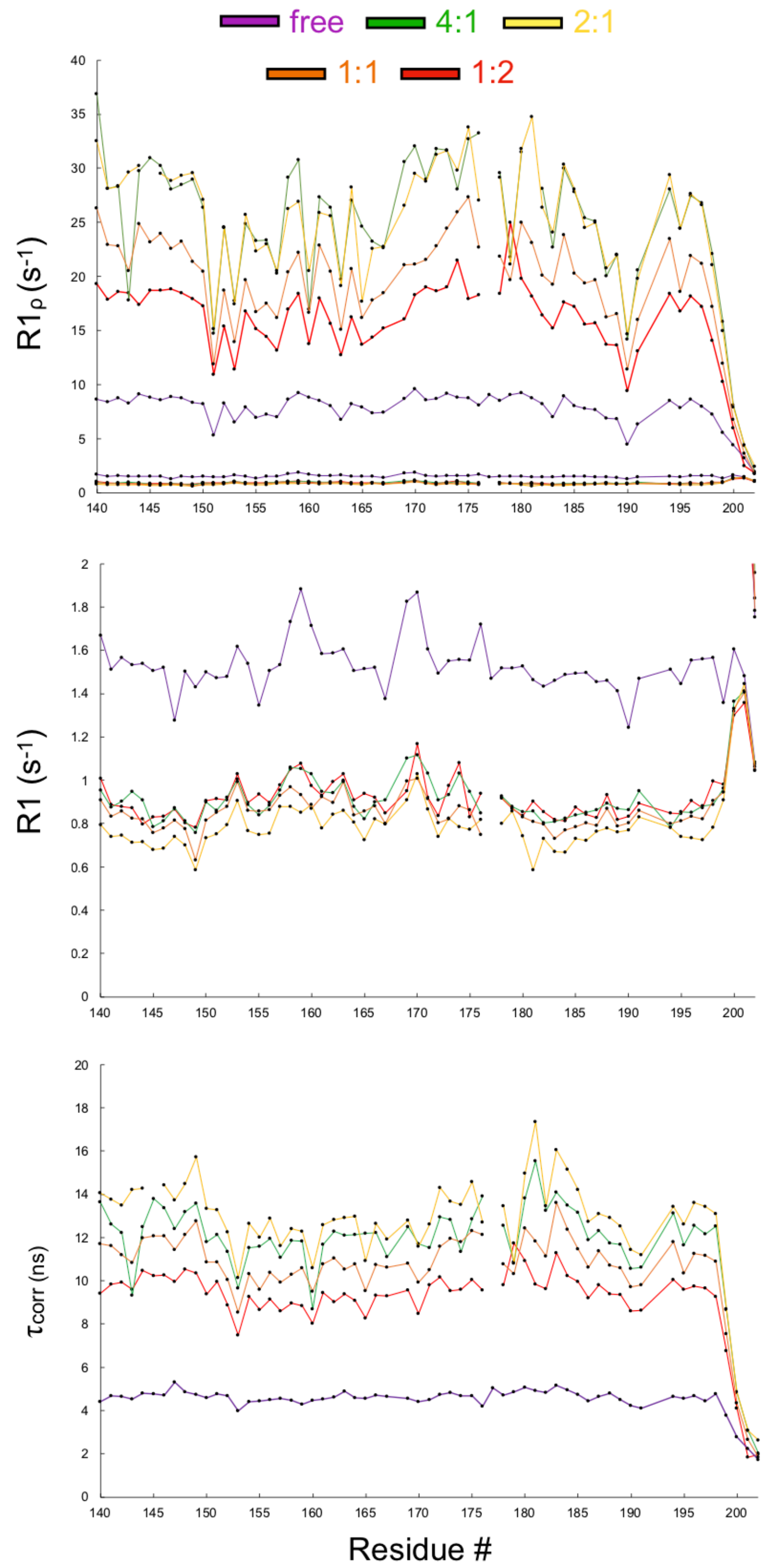

Supplementary Figure S10. ^{15}N NMR relaxation measurements of the Zα:AluSx1Jo RNA complex at different RNA concentrations. Average rotating-frame relaxation rates ($R_{1\rho}$, top), longitudinal (R_1, middle), and the extracted effective overall correlation times (τ_{corr}, bottom) are shown for the Zα:AluSx1Jo RNA ratios indicated. All relaxation rates were measured at 900 MHz.

(a)

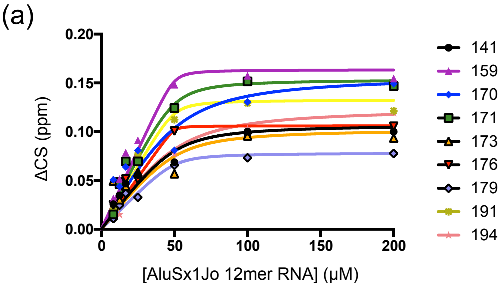

(b)

Residue #	K_D (μM)
141	4.40 ± 5.80
159	0.30 ± 0.85
170	9.15 ± 12.48
171	1.70 ± 2.18
173	5.19 ± 11.8
176	0.11 ± 0.74
179	1.48 ± 2.19
191	0.67 ± 1.83
194	8.90 ± 6.15
Global	2.04 ± 1.73

(c)

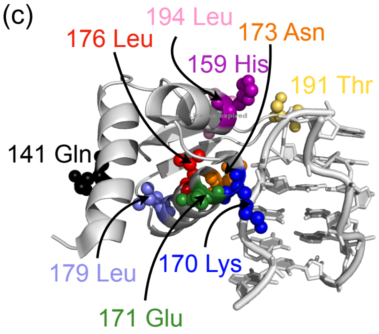

Supplementary Figure S11. Individual dissociation constant (K_d) fits from the ^{15}N-HSQC titration of AluSx1Jo RNA into Zα. (a) The residues which showed significant chemical shift perturbations (CSPs) from the ^{15}N-HSQC titration of AluSx1Jo RNA into Zα were fit to a quadratic binding equation assuming a two-site binding model (see **Methods** for more details). The change in the chemical shift is plotted on the y-axis versus the concentration of AluSx1Jo RNA ligand on the x-axis. (b) Individual K_d values for each residue. (c) Atoms of the residues used in K_d analysis are plotted onto a ribbon representation of the crystal structure of Zα (PDB: 3f21).

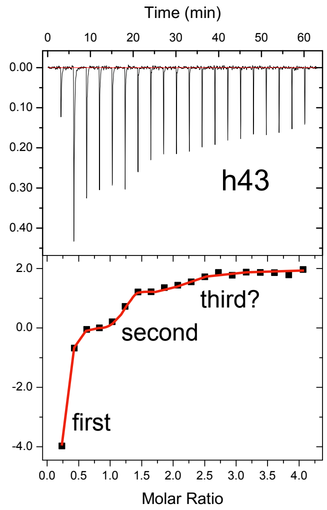

N1: 0.3 ± 0.0
N2: 1.1 ± 0.0
N3: 2.1 ± 0.0

K_D1: 60.0 ± 150.0 nM
K_D2: 90.1 ± 153.4 nM
K_D3: 1540.0 ± 3370.0 nM

ΔH1: -4.4 ± 0.2 kcalmol^{-1}
ΔH2: -1.3 ± 57.0 kcalmol^{-1}
ΔH3: -0.1 ± 0.1 kcalmol^{-1}

TΔS1: 5.5 kcalmol^{-1}
TΔS2: 8.3 kcalmol^{-1}
TΔS3: 6.9 kcalmol^{-1}

ΔG1: -9.9 kcalmol^{-1}
ΔG2: -9.6 kcalmol^{-1}
ΔG3: -7.9 kcalmol^{-1}

Supplementary Figure S12. **Isothermal calorimetry thermogram from injection of h43 *E. coli* into Zα**. The ITC thermogram is shown the and the fitted parameters on the right.

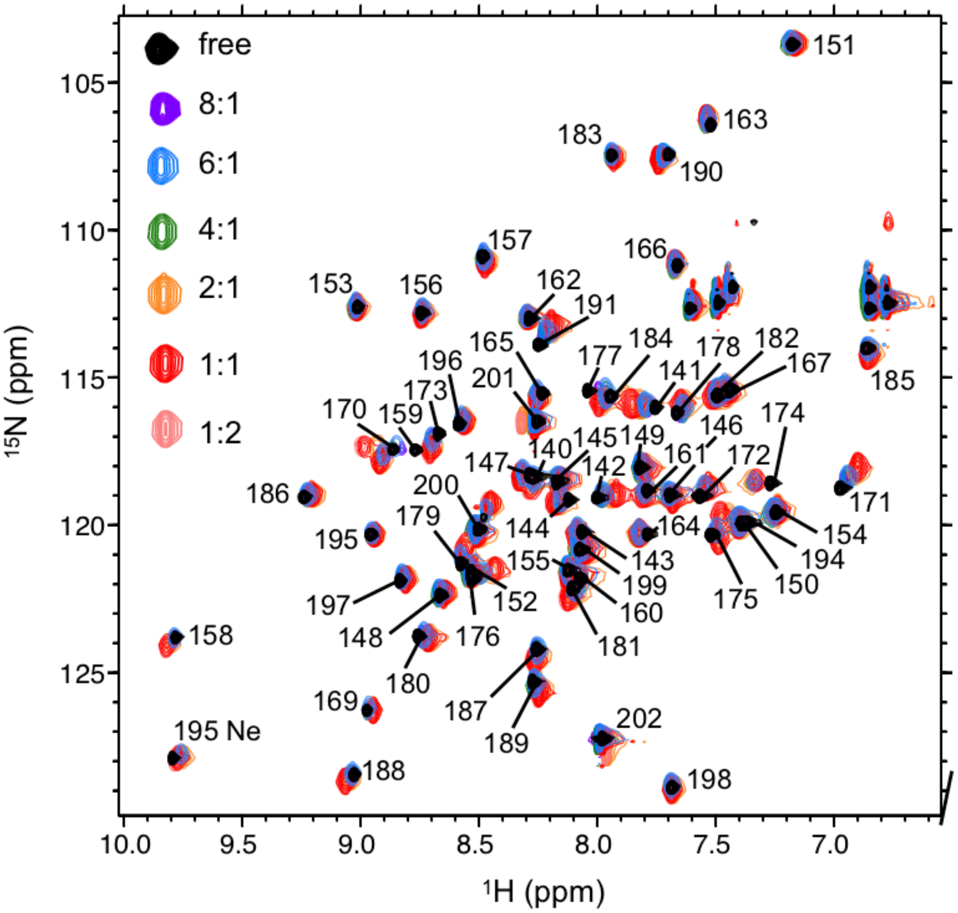

Supplementary Figure S13. Full Zα ^{15}N-HSQC upon addition of AluSx1Jo . The full ^{15}N-HSQC spectra at each point of the Zα:AluSx1Jo RNA titration and peak assignments are shown. The ratio of Zα:AluSx1Jo RNA and its corresponding color is indicated.

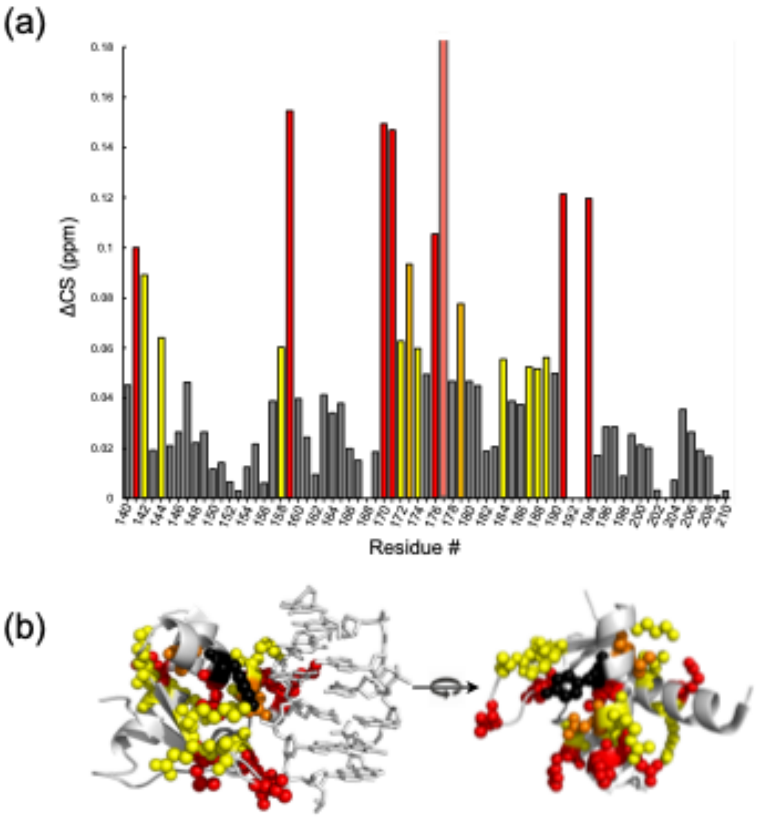

Supplementary Figure S14. Residue-specific CSPs from of binding of Zα to AluSx1Jo. (a) Difference in the ^{15}N-HSQC chemical shifts between the free and bound (free vs 1:2 Zα:AluSx1Jo). Differences greater than 0.1 ppm are colored red, between 0.090 and 0.099 are orange, and those between 0.060 and 0.089 are yellow. Tyrosine 177 is marked with a faint red bar to indicate that it broadened beyond detection early into the titration, so its maximal chemical shift change is unknown. (b) CSPs from (a) are plotted onto the structure of Zα bound to d(CpG)$_3$ RNA (PDB: 3f21). Tyrosine 177 is colored black to indicate that binding to AluSx1Jo causes it to disappear from the spectrum early in the titration.

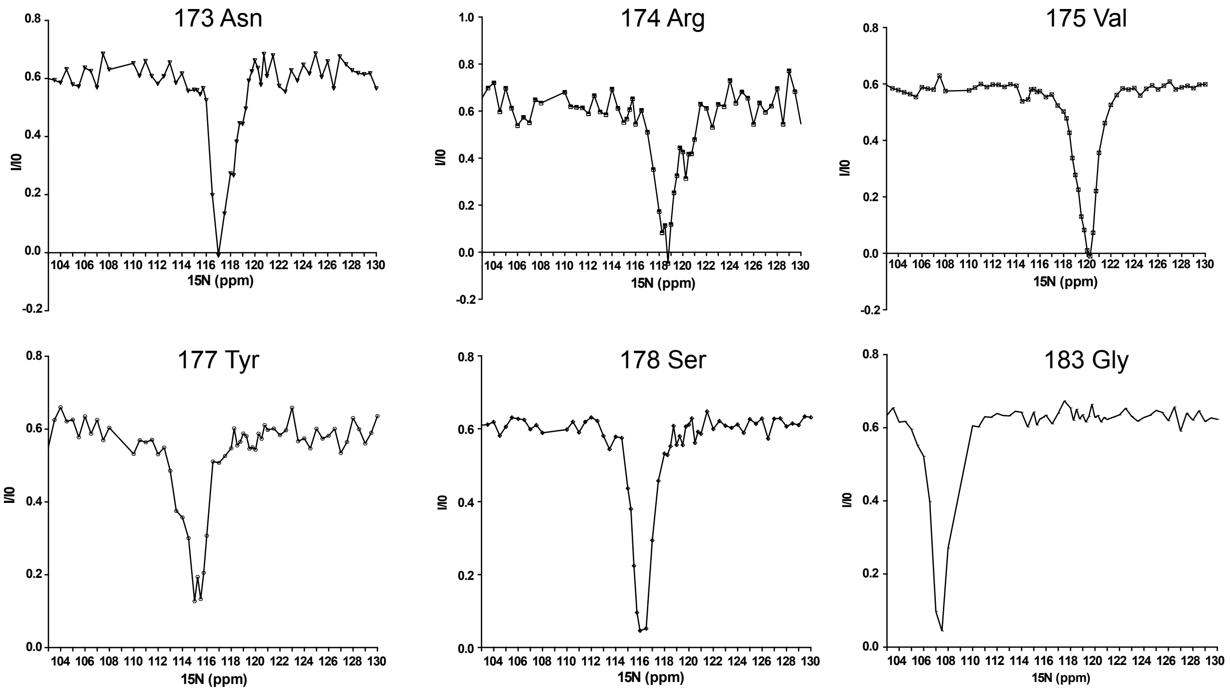

Supplementary Figure S15. Chemical Exchange Saturation Transfer profiles for Zα binding residues at 10:1 Zα:AluSx1Jo. CEST profiles of asparagine 173, arginine 174, valine 175, tyrosine 177, serine 178, and glycine 183 (as a negative control) are shown.

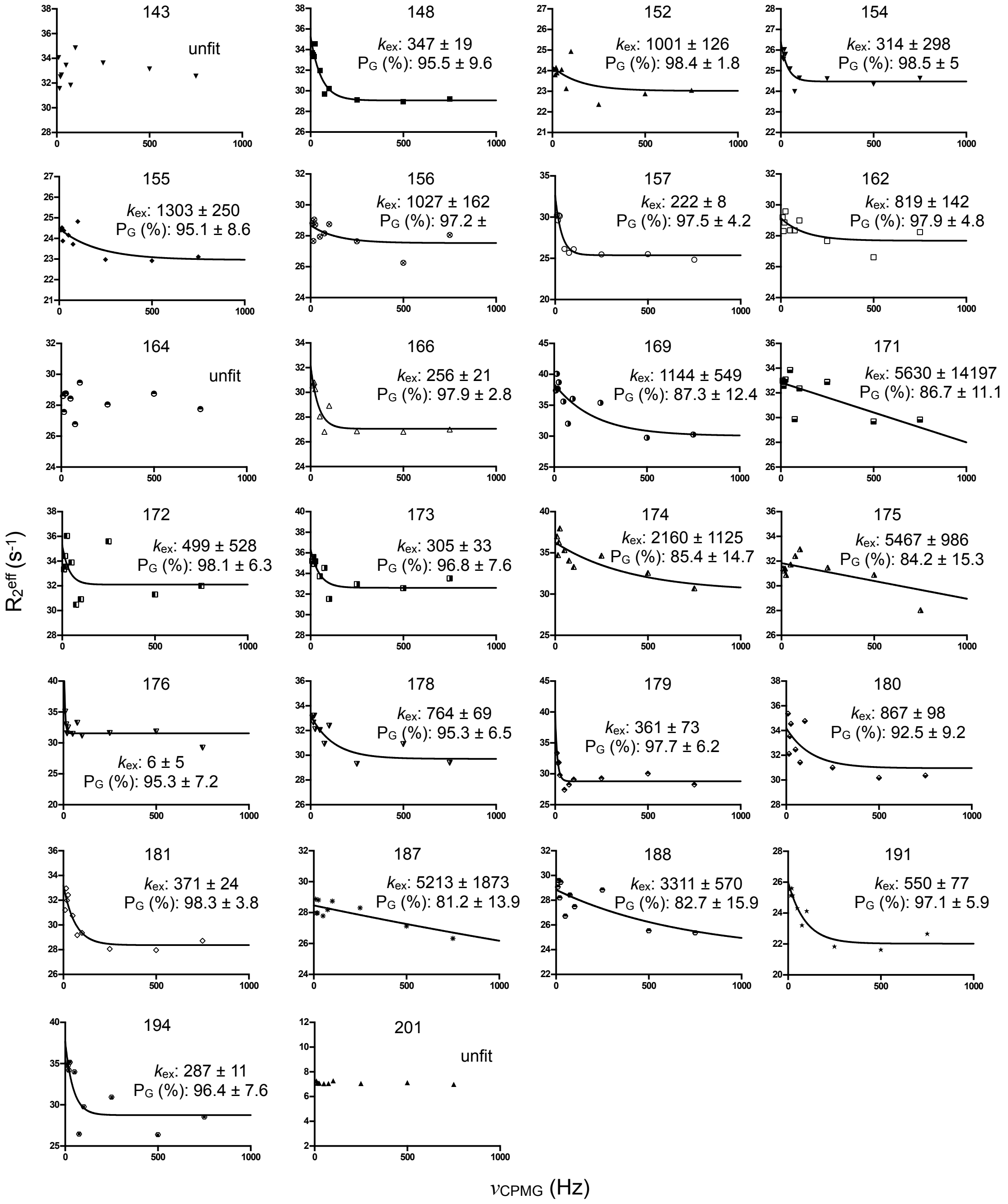

Supplementary Figure S16. Carr-Purcell-Meiboom-Gill (CPMG) relaxation dispersion of Zα bound to the AluSx1Jo RNA. CPMG relaxation dispersion profiles measured at 2:1 Zα: AluSx1Jo RNA representing a fully saturated complex. The global k_{ex} across all residues is 634 s^{-1} and the major state population is 96.2 ± 6.4 %, and for the binding helix 770 s^{-1} with a major state population of 95.5 ± 7.5 %. Values are tabulated in Supplementary Table S2. For details about the fits, see Materials and Methods.

Supplementary Table S1. E_Z Scores for Different RNA Sequence Contexts.

Helix Identity	Species	E_Z Score (Zα)	E_Z Score ($Zα_{Tyr177Ala}$)
$G_{10}C_{10}$	N/A	0.04	
$(ApU)_6$		0.04	
14mer cUUCGg tetraloop	N/A	-0.03	
14mer cGAAAg tetraloop	N/A	0.03	
AluSx1Jo (capped)	*H.sapiens*	0.10	
H66*	*H.sapiens*	0.12	
H25	*E.coli*	0.13	
h30	*H.sapiens*	0.14	
h30	*E.coli*	0.15	
h41	*H.sapiens*	0.15	
h43	*E.coli*	repeat 1: 0.10<br>repeat 2: 0.16	-0.07
A-Z 2	N/A	0.19	-0.02
A-Z 1	N/A	0.20	-0.11
H66	*E.coli*	0.30	
AluSx1Jo	*H.sapiens*	0.32	-0.04
H66 35mer*	*H.sapiens*	0.43	
h41*	*E.coli*	0.47	
$(CpG)_6$	N/A	0.90	
$(CpG)_3$	N/A	1.0	0.60

E_Z scores should be taken with an error of 0.1 based on repeat measurements of h43 *E.coli* with Zα and the difference between $(CpG)_3$ and $(CpG)_6$. *Indicates duplex formation when stem-loop was expected.

Supplementary Table 2. Kinetic Parameters Extracted from Carr-Purcell-Meiboom-Gill Measurements Fits.

Residue #	$R_{2,0}$ (s^{-1})	k_{ex} (s^{-1})	P_G (%)
148	29 ± 0.1	347 ± 19	95.5 ± 9.6
152	23.0 ± 0.1	1001 ± 126	98.4 ± 1.8
154	24.5 ± 0.2	314 ± 298	98.5 ± 5.0
155	22.9 ± 0.1	1303 ± 250	95.1 ± 8.6
156	27.5 ± 0.1	1027 ± 162	97.2 ± 4.5
157	25.3 ± 0.1	222 ± 8	97.5 ± 4.2
162	27.6 ± 0.1	819 ± 142	97.9 ± 4.8
166	27.0 ± 0.1	256 ± 21	97.9 ± 2.8
169	30.0 ± 0.8	1144 ± 549	87.3 ± 12.4
171	15.5 ± 9.9	5630 ± 14197	86.7 ± 11.1
172	32.4 ± 0.5	499 ± 528	98.1 ± 6.3
173	32.6 ± 0.1	305 ± 33	96.8 ± 7.6
174	30.4 ± 1.0	2160 ± 1125	85.4 ± 14.7
175	27.5 ± 0.6	5467 ± 986	84.2 ± 15.3
176	31.5 ± 0.1	6 ± 5	95.3 ± 7.2
178	29.6 ± 0.1	764 ± 69	95.3 ± 6.5
179	28.7 ± 0.1	361 ± 73	97.7 ± 6.2

180	30.9 ± 0.1	867 ± 98	92.5 ± 9.2
181	28.3 ± 0.1	371 ± 24	98.3 ± 3.8
187	25.0 ± 1.0	5213 ± 1873	81.2 ± 13.9
188	24.2 ± 0.4	3311 ± 570	82.7 ± 15.9
191	22.0 ± 0.1	550 ± 77	97.1 ± 5.9
194	28.6 ± 0.1	287 ± 11	96.4 ± 7.6

Supplementary Table S3. Kinetic Parameters Extracted from Global Carr-Purcell-Meiboom-Gill Measurements Fit.

Residue #	$R_{2,0}$ (s^{-1}) global fit	$R_{2,0}$ (s^{-1}) global fit (binding site)	k_{ex} (s^{-1}) global fit	k_{ex} (s^{-1}) global fit (binding site)	P_G (%) global fit	P_G (%) global fit (binding site)
148	29.1 ± 0.8		633 ± 0		96.2 ± 6.4	
152	22.9 ± 0.6		633 ± 0		96.2 ± 6.4	
154	24.1 ± 0.6		633 ± 0		96.2 ± 6.4	
155	22.9 ± 0.6		633 ± 0		96.2 ± 6.4	
156	27.2 ± 0.5		633 ± 0		96.2 ± 6.4	
157	25.4 ± 0.8		633 ± 0		96.2 ± 6.4	
162	27.4 ± 0.7		633 ± 0		96.2 ± 6.4	
166	27.6 ± 0.5		633 ± 0		96.2 ± 6.4	
169	31.4 ± 1.5	30.8 ± 1.3	633 ± 0	771 ± 0	96.2 ± 6.4	95.5 ± 7.5
171	29.9 ± 0.9	29.9 ± 0.8	633 ± 0	771 ± 0	96.2 ± 6.4	95.5 ± 7.5
172	31.6 ± 0.8	31.8 ± 0.5	633 ± 0	771 ± 0	96.2 ± 6.4	95.5 ± 7.5
173	32.3 ± 0.9	32.3 ± 0.5	633 ± 0	771 ± 0	96.2 ± 6.4	95.5 ± 7.5
174	32.1 ± 1.0	31.9 ± 0.9	633 ± 0	771 ± 0	96.2 ± 6.4	95.5 ± 7.5
175	30.0 ± 0.7	30.1 ± 0.6	633 ± 0	771 ± 0	96.2 ± 6.4	95.5 ± 7.5
176	30.8 ± 0.8	30.9 ± 0.6	633 ± 0	771 ± 0	96.2 ± 6.4	95.5 ± 7.5
178	29.7 ± 1.0	28.4 ± 0.4	633 ± 0	771 ± 0	96.2 ± 6.4	95.5 ± 7.5
179	28.6 ± 0.6	28.7 ± 0.5	633 ± 0	771 ± 0	96.2 ± 6.4	95.5 ± 7.5
180	31.0 ± 0.8	31.1 ± 0.5	633 ± 0	771 ± 0	96.2 ± 6.4	95.5 ± 7.5
181	28.5 ± 0.7		633 ± 0		96.2 ± 6.4	
187	26.8 ± 0.7		633 ± 0		96.2 ± 6.4	
188	25.9 ± 0.9		633 ± 0		96.2 ± 6.4	
191	22.1 ± 0.8	22.3 ± 0.4	633 ± 0	771 ± 0	96.2 ± 6.4	95.5 ± 7.5
194	28.8 ± 1.0		633 ± 0		96.2 ± 6.4	
