# Supplementary material for: Recognition of non-CpG repeats in Alu and ribosomal RNAs by the Z-RNA binding domain of ADAR1 induces A-Z junctions
Source: Nat Commun. 2021 Feb 4;12:793. doi: 10.1038/s41467-021-21039-0 (PMC7862695; doi:10.1038/s41467-021-21039-0)
Supplement: Supplementary file 3 — Reporting Summary [file 41467_2021_21039_MOESM3_ESM.pdf]

nature research

Corresponding author(s): Quentin Vicens, Beat Vögeli

Last updated by author(s): December/31/2020

Reporting Summary

Nature Research wishes to improve the reproducibility of the work that we publish. This form provides structure for consistency and transparency in reporting. For further information on Nature Research policies, see our Editorial Policies and the Editorial Policy Checklist.

Statistics

For all statistical analyses, confirm that the following items are present in the figure legend, table legend, main text, or Methods section.

n/a	Confirmed	
☐	☒	The exact sample size (*n*) for each experimental group/condition, given as a discrete number and unit of measurement
☐	☒	A statement on whether measurements were taken from distinct samples or whether the same sample was measured repeatedly
☐	☒	The statistical test(s) used AND whether they are one- or two-sided<br>*Only common tests should be described solely by name; describe more complex techniques in the Methods section.*
☒	☐	A description of all covariates tested
☐	☒	A description of any assumptions or corrections, such as tests of normality and adjustment for multiple comparisons
☐	☒	A full description of the statistical parameters including central tendency (e.g. means) or other basic estimates (e.g. regression coefficient) AND variation (e.g. standard deviation) or associated estimates of uncertainty (e.g. confidence intervals)
☒	☐	For null hypothesis testing, the test statistic (e.g. *F*, *t*, *r*) with confidence intervals, effect sizes, degrees of freedom and *P* value noted<br>*Give P values as exact values whenever suitable.*
☒	☐	For Bayesian analysis, information on the choice of priors and Markov chain Monte Carlo settings
☒	☐	For hierarchical and complex designs, identification of the appropriate level for tests and full reporting of outcomes
☐	☒	Estimates of effect sizes (e.g. Cohen's *d*, Pearson's *r*), indicating how they were calculated

Our web collection on statistics for biologists contains articles on many of the points above.

Software and code

Policy information about availability of computer code

Data collection	VNMRJ version 4.2 Revision A (Agilent), TopSpin version 7 (Bruker), ProteomeLab version 6.0 (Beckman), Spectra Manager 2 (JASCO), ITC 200 version 1.26.1 (Malvern)
Data analysis	ccpNMR analysis version 2.4.2 (CCPN), NMRpipe version 10.9 (NIST IBBR), SEDFIT version 14.7g (NIH), Microcal Analysis version 7 SR4 (Origin)

For manuscripts utilizing custom algorithms or software that are central to the research but not yet described in published literature, software must be made available to editors and reviewers. We strongly encourage code deposition in a community repository (e.g. GitHub). See the Nature Research guidelines for submitting code & software for further information.

Data

Policy information about availability of data

All manuscripts must include a data availability statement. This statement should provide the following information, where applicable:

- Accession codes, unique identifiers, or web links for publicly available datasets
- A list of figures that have associated raw data
- A description of any restrictions on data availability

The datasets generated during and/or analyzed during the current study are available in the Dryad repository, with the identifier doi:10.5061/dryad.pvmcvdnk4. The crystal structures used in Figures 6 and 7 are available online from RCSB at the following links:
2GXB: https://www.rcsb.org/structure/2GXB
5ZUO: https://www.rcsb.org/structure/5ZUO
3F21: https://www.rcsb.org/structure/3F21

Field-specific reporting

Please select the one below that is the best fit for your research. If you are not sure, read the appropriate sections before making your selection.

- [x] Life sciences
- [ ] Behavioural & social sciences
- [ ] Ecological, evolutionary & environmental sciences

For a reference copy of the document with all sections, see nature.com/documents/nr-reporting-summary-flat.pdf

Life sciences study design

All studies must disclose on these points even when the disclosure is negative.

Sample size	A total of 25 RNA fragments were included in this study (incl. 2 positive controls, and 4 negative controls). RNAs were chosen based off previous literature results suggesting possible Z-RNA formation, and positive/negative controls were added as needed. No statistical methods were used to chose the sample size.
Data exclusions	No data were excluded from the analyses.
Replication	CD measurements were performed 1X (samples), or 2X (controls and h43 and AluSx1Jo). NMR measurements were performed once as customary, and showed consistency with CD, ITC and AUC experiments.
Randomization	Not applicable. RNAs were chosen specifically for their previously identified ability to either bind to Za or adopt the Z-conformation. RNAs were measured usually one or two at a time over the course of the study.
Blinding	Blinding is not relevant to this study as the experimenter always knew what RNA sequence was being examined. The main conclusion of the paper was not apparent until all RNA fragments had been analyzed.

Reporting for specific materials, systems and methods

We require information from authors about some types of materials, experimental systems and methods used in many studies. Here, indicate whether each material, system or method listed is relevant to your study. If you are not sure if a list item applies to your research, read the appropriate section before selecting a response.

Materials & experimental systems

n/a	Involved in the study	
x		Antibodies
x		Eukaryotic cell lines
x		Palaeontology and archaeology
x		Animals and other organisms
x		Human research participants
x		Clinical data
x		Dual use research of concern

Methods

n/a	Involved in the study	
x		ChIP-seq
x		Flow cytometry
x		MRI-based neuroimaging
