# Supplementary material for: Recognition of non-CpG repeats in Alu and ribosomal RNAs by the Z-RNA binding domain of ADAR1 induces A-Z junctions
Source: Nat Commun. 2021 Feb 4;12:793. doi: 10.1038/s41467-021-21039-0 (PMC7862695; doi:10.1038/s41467-021-21039-0)
Supplement: Supplementary file 2 — Peer Review File [file 41467_2021_21039_MOESM2_ESM.pdf]

Reviewer #1 (Remarks to the Author):

A to I editing is a form of post-transcriptional RNA modification carried out by the ADAR family of proteins, that plays important roles in immune response and self vs. non-self RNA recognition. The structural basis for recognition of a wide variety of sequences by ADAR1 is unknown. In this study, through an elegant combination of multiple biophysical techniques the authors show that Zα domain of ADAR1 can bind a variety of RNA sequences, with varying propensities which is related to their ability to adopt the Z-form conformation. The manuscript is suitable for publication in Nat Comm once the authors address the following comments.

Major comments

• Regarding the Ez score metric used by the authors –can the authors comment on the importance of including the decrease in ellipticity at 266nm in defining Ez. In a hypothetical scenario, two RNAs containing A-Z junctions with exactly the same Z-form shape, but different deviations from the idealized A-form shape in regions neighboring the Z-form section, would have different Ez scores, even though the Z-form context is the same? Is the above scenario possible?
• Figures 2b and c, AluSx1Jo duplex – The values of ellipticity at 285 nm and 295 nm for the WT protein-RNA complex are similar to that for the unbound RNA, while those for the Tyr177Ala mutant are higher than that for unbound RNA – Wouldn't this suggest that the Z form content in the mutant protein-RNA complex is higher than wild-type? In addition, examination of the 266nm values would suggest that the A-form content is less in the wild-type protein-RNA complex relative to the mutant? However, the interpretation using the Ez scores would suggest that the Z-form content in the mutant protein is less than that in wild-type protein, likely cause it penalizes the decrease in intensity in 266nm values in the wild-type (also see point below). The same argument can also be made for the h43 construct. Please clarify
• There is also a dip in the CD intensity at ~290nm prominent at higher concentrations of wild-type protein (Figure S2), which is close to the reporter wavelength of 295nm used in the Ez score definition – the authors should clarify how/why this dip does not affect their interpretation of the data (eg. AluSx1Jo shows dips at 290nm at high Zα concentration, that could lead to under-estimation of the Z-form content). A concentration dependent CD titration of the Tyr177Ala mutant would also be useful to include here.
• Is there evidence to rule out that changes are CD are due to conformational changes of the protein on binding to RNA?

Although not essential for this study, the authors may want to think about correlating editing levels of ADAR1 sites in RNA (ref. 12) with the predicted secondary structures of these sites, a correlation similar to Figure S4b, between editing levels and non-canonical base pairs could be observed.

• Could helix destabilizing regions marked in Figure 3 actually be regions on the RNA wherein the protein binds, but the imino peaks are lost due to exchange broadening? Could the peaks that show CSPs be regions neighboring the Z-form portion in the bound form, in fast exchange between 2 conformations? Is there a way to rule out this explanation for the data?
• For example, AUC data on h43 would suggest a 4:1 complex being formed. However, the current Z-alpha binding site (Figure 3b) only spans 7 base pairs, coupled with the fact that a given protein binding site spans 5 base pairs, wouldn't this suggest that only two protein molecules can bind? Could there be 2 binding sites spanning bases 1-5 and 6-10 instead? Or is there only one binding site with non-specific binding of 2 additional proteins? Please clarify.
• Similar arguments can also be applied to the H25 construct. The current binding site only has 5 base pairs, while the AUC data suggests a 1:4 complex. Could there be 2 binding sites instead from base

pairs 3-7 and 8-12?
• Also, for the H66 construct, could there be 2 binding sites from 3-7 and 8-12? Given that the helix destabilizing region actually has many pyrimidine-purine steps conducive for Z-form formation, could 2 protein molecules not bind in the middle of the RNA?

Minor comments

• Potential typo in sentence 1 "… adenosine deaminase that acts on RNA 1 (ADAR1) …", Should this be changed to "… adenosine deaminase that acts on RNA (ADAR1) …"?
• It would be instructive to add amino acid residue numbering in Figure 1a, to enable readers to gauge the size of the domains and linkers.
• Does A to I editing by ADAR1 in Alu repeats occur in non CpG regions? It would be instructive to clarify this somewhere in the introduction.
• It would be instructive to show interactions formed by Lys169 with CpG RNA in Figure 1c, and position of residue 141 in Figure 6c, as these have key interactions with RNA/show large CSPs on RNA binding.
• Methods section: Circular Dichroism– "A control experiment was run to ensure that the absorbance of Zα alone in the 220-320 nm range at the same concentrations was minimal (Supplementary Figure S2)" - Examination of Supplementary Figure S2 suggests that absorbance of Zα alone does change from 220-250nm by an amount comparable to changes in absorbance in Zα+RNA samples – please clarify. The authors should also add y and x axes labels to Figure S2.
• Methods section: ITC experiments – The authors should provide the two-site equation to used to fit ITC data in Figure 5a, in addition to mentioning how errors in Table 1 were computed.
• Methods section: Analytical centrifugation – Please provide a reference that describes fitting of the data.
• Methods section: NMR experiments – "The 2D 1H-1H NOESY recorded for H66 E. coli …" – Should this be changed to H66 H. sapiens?
• Methods section: Kd fitting for titration points – The authors should explain how they obtained the factor of 0.2 in front of the 15N chemical shift differences in the formula for computing the CSP. Should the factor not be γH/γN instead, where γ is the gyromagnetic ratio? The authors should also provide a derivation for the equation for estimating Kds from CSPs and mention any assumptions involved in its derivation, or refer to a prior study with this information.
• Figures 2b and c – Please use the same y-axis scales for the AluSx1 and h43 samples. This would facilitate easy comparison of the effects of the mutation.
• Figure 2e – Please describe how the fractional decrease was computed in methods.
• Figure S5 – For the panels on the right, please provide the x and y axes labels, in addition to explaining insets in the panels (please also do so for Figure 5e). The authors should also provide some sense of the errors in the AUC values and explain how band assignments were made. For example, for H25 E. Coli the major peak in the AUC data is at 36kDa, while the predicted weight for the assigned 1:4 complex is 39.1 kDa. Mentioning the complex stoichiometry next to the peak molecular weight in the AUC data would also be useful.
• Figure 7 and "The binding of Zα to AluSx1Jo is more dynamic than for (CpG)3 RNA" section – Were the comparisons of kex between (CpG)3 DNA, (CpG)3 RNA and AluSx1Jo RNA made for similar populations of the bound and unbound forms? In all cases, exchange is between free and 2:1 (protein:RNA) complexes, right? Would be instructive to clarify this.
• Figure 5b - How were averages for R1, R1p and tau_corr computed for this figure. Can the set of numbers be used to get error bars for R1, R1p and tau_corr? Also, the authors should comment on why is correlation time for the 4:1 complex is less than that for the 2:1 complex, while the expectation based on the stoichiometry would suggest otherwise?
• Results: "A-Z junction adoption in AluSx1Jo, H66, h43, and H25 involves destabilization of adjacent A-RNA" section, Page 11 – "For H25, Zα appeared to target the region in the middle of the stem which contained only one (YpR) step and two G.G base pairs (Figure 3d)." – should this be G.A mispairs instead of G.G?
• Results: "Zα binds cooperatively to AluSx1Jo and h43 in the ~500 nM–2 μM range" section – "These

tumbling times were consistent with a 2:1 Zα:AluSx1Jo complex being formed at a 2:1 protein:RNA molar ratio, a 1:1 complex at 1:2 protein:RNA, and an intermediate complex between 2:1 and 1:1 at a 1:1 molar ratio of protein:RNA" – I would suggest re-wording this sentence to – "These tumbling times were consistent with a 2:1 Zα:AluSx1Jo complex being formed at a 2:1 protein:RNA molar ratio, an intermediate complex between 2:1 and 1:1 at a 1:1 molar ratio of protein:RNA and a 1:1 complex at 1:2 protein:RNA", for ease of readability.

Reviewer #2 (Remarks to the Author):

This paper reports on biophysical study of the A-Z junction formation of various RNA induced by Zα protein. The authors proposed that Zα proteins bind to a CG-rich region in the non-CG-repeat RNA via novel mechanism which combines the CG-repeat DNA binding model and B-Z junction formation model. The results are of interesting and the experimental data and controls look convincing. Therefore, I recommend publication almost in the present form, but would suggest that the authors revised as considering the following:

1) The authors estimated the relative Z-contents by Ez from CD data and the Z-RNA forming and A-Z junction base-pairs by imino proton spectra. These approaches are of interest and could be applicated in other study of the nucleic acid-protein interactions. The authors determined the molar ratios of Zα:RNA complexes by AUC. Generally, two Zα molecules span 4~6 base-pairs via binding to each strand of RNA). For example, the Zα:RNA complex with 4:1 molar ratio contains at least 8-bp Z-RNA (possibly 9~10 bp). The contents of Z-RNA base-pair might be correlate with Ez value. Thus, I suggest the authors discuss more the correlation between these parameters.

2) In addition, in Figure 3, when the Zα binding region is determined, the Zα:RNA molar ratio and Ez value are should be considered. In Discuss section, if you mention that, the reader can understand your concept more easily.

3) I cannot find the AUC data for the AluSx1Jo RNA in Figure S5.

4) When you mentioned Z-DNA binding model, the original paper about this model (Kang et al., JACS 131 (2009), 11485.) should be sited.

Reviewer #3 (Remarks to the Author):

Nichols and coworkers investigate the interaction between the Z-DNA binding domain of ADAR1 (Zalpha) and non-CpG repeats of various RNAs, e.g. Alu and rRNA. The authors show by a variety of techniques that a broad range of RNA sequences bind to Zalpha adopting a Z-RNA conformation, and being accompanied by a simultaneous destabilization of neighboring A-form regions. The knowledge on Zalpha (and other) Z-RNA inducing protein domains is very scarce and only at the very beginning of grasping its importance. In addition, this study is carefully conducted, and the results based on a broad variety of experiments. Hence, I can recommend this manuscript for publication in Nature Commun, taking the below minor comments into consideration first.
i) In the last sentence of the abstract the authors highlight the implication of their findings in the role of Zalpha with respect the attack of a pathogen on a cell. Such a statement should be made with care because in the final conclusion section, it becomes evident that basically all implications for any role of Zalpha are still hypothetical, with no real evidence (yet), e.g. typical phrases used are

"if/could/would/remains unclear …" etc. This does by no means diminish the importance of the findings, but the authors should simply stick to the facts, and not imply anything that has not been shown. The results themselves are important enough. Please adjust.
ii) page 5, calculation of Ez; it is unclear where the factors 1.8, 0.718, and 1.109 in the given equation come from. Please explain.
iii) page 7, fitting of NMR titrations: the CSP is calculated from the average of 1H and 15N chemical shift changes. Why? It would be much better to fit the CSP of 1H and 15N individually and take the average. Also the factor 0.2 is not explained. In this whole section no reference is given, please add. The second equation provided (obviously coming from a quadratic equation) should be derived/explained in more detail. Such CSPs go back to the 70ies, originally derived for UV measurements (same dependence as NMR shifts), and have been adopted subsequently, see e.g. Mitchell in J Chem Soc Dalton Trans (1979) 771-776, and then slightly adopted, e.g. in Helv Chim Acta (1995) 78 : 1112-1143 or JBIC (2000) 5 : 287-299.

- We wish to thank all reviewers for their positive and insightful comments, to which we respond below, one by one.

Reviewer 1

Major comments

- Regarding the Ez score metric used by the authors –can the authors comment on the importance of including the decrease in ellipticity at 266nm in defining Ez. In a hypothetical scenario, two RNAs containing A-Z junctions with exactly the same Z-form shape, but different deviations from the idealized A-form shape in regions neighboring the Z-form section, would have different Ez scores, even though the Z-form context is the same? Is the above scenario possible?

- Our method is based off of the assumption that the RNA in question starts off predominately in the A-form conformation, and if this is not the case, the approximation is not as rigorous. We consider not only the changes in the molar ellipticities at 285 and 295 nm, but also at 266 nm because the loss of the A-form peak is directly related to the transition to the Z-conformation. For Z-RNA junctions where only a portion of the RNA adopts the Z-conformation, the growth at 285 and 295 is not as extensive compared to the (CpG)n repeat (as was the case for A-Z 1 and A-Z 2, Figure 2), making including the decrease at 266 nm more important. However, this also means that the E_Z score is sensitive to both the switch from the A- to the Z-conformation and also the destabilization of the neighboring A-RNA.

-We have added the above description to the methods section.

- Figures 2b and c, AluSx1Jo duplex – The values of ellipticity at 285 nm and 295 nm for the WT protein-RNA complex are similar to that for the unbound RNA, while those for the Tyr177Ala mutant are higher than that for unbound RNA – Wouldn’t this suggest that the Z form content in the mutant protein-RNA complex is higher than wild-type? In addition, examination of the 266nm values would suggest that the A-form content is less in the wild-type protein-RNA complex relative to the mutant? However, the interpretation using the Ez scores would suggest that the Z-form content in the mutant protein is less than that in wild-type protein, likely cause it penalizes the decrease in intensity in 266nm values in the wild-type (also see point below). The same argument can also be made for the h43 construct. Please clarify

-While the Za mutant does not induce the Z-conformation, it can still bind to RNA and appears to stabilize the A-form content, causing an increase to the molar ellipticity at 266, and since the 266 peak is the major peak, causes indirect increases at 285 and 295 as well. This is one reason why it is important to include the decrease in the molar ellipticity at 266 nm. We have adjusted the methods section so that is now explained.

- There is also a dip in the CD intensity at ~290nm prominent at higher concentrations of wild-type protein (Figure S2), which is close to the reporter wavelength of 295nm used in the Ez score definition – the authors should clarify how/why this dip does not affect their interpretation of the data (eg. AluSx1Jo shows dips at 290nm at high Z☐ concentration, that could lead to under-estimation of the Z-form content). A concentration dependent CD titration of the Tyr177Ala mutant would also be useful to include here.

-We have measured and added the CD titration of Tyr177Ala alone to Figure S2, and now mention this measurement in the methods section.
-Yes, due to the minor negative molar ellipticity at 290 nM by Za alone, our Z-score may underestimate the extent of Z-conformation. However, since the E_Z score is weighted by the (CpG)3 repeat, which has the same concentration of Za as the other test RNAs, this contribution is mostly canceled out. We have added the following text to the methods: "While the E_Z score may be slightly underestimated by negative contribution to the molar ellipticity at ~ 290 nm at the higher concentrations of Zα (Figure S2), this contribution is relatively small and is mostly canceled out by the fact that we weight the E_Z score according to the (CpG)3 repeat (which has the same concentration of Zα as the other constructs)."

- Is there evidence to rule out that changes are CD are due to conformational changes of the protein on binding to RNA?

-There are minimal chemical shift changes to Za upon titration of RNA, suggesting that the protein has minimal conformation changes upon binding. We have adjusted the following sentence: "Together, these three changes were indicative of a conversion to the Z-conformation [22], and are unlikely to be due to a conformational change in Zα upon binding RNA as our ^{15}N-HSQC titration indicates binding only results in minor rearrangements to the RNA binding residues of Zα (Supplemental Figure S12, S13)."
-In addition, absorbance in the 250-320 range in CD for a protein is rare.

Although not essential for this study, the authors may want to think about correlating editing levels of ADAR1 sites in RNA (ref. 12) with the predicted secondary structures of these sites, a correlation similar to Figure S4b, between editing levels and non-canonical base pairs could be observed.

-We could not agree more, and we are actually in the process of working on this, which will be described in an upcoming manuscript.

- Could helix destabilizing regions marked in Figure 3 actually be regions on the RNA wherein the protein binds, but the imino peaks are lost due to exchange broadening? Could the peaks that show CSPs be regions neighboring the Z-form portion in the bound form, in fast exchange between 2 conformations? Is there a way to rule out this explanation for the data?

-We cannot rule out that it is due to exchange broadening, however, the extreme similarity to the B-Z DNA titration suggests this is not the case. In addition, in the case of intermediate exchange we would expect for these peaks to reemerge at higher concentrations of Za which was not the case. We have added the following sentence: "While the observed disappearance of imino peaks could be caused by line broadening due to intermediate exchange, the striking similarity to titrations done on the B-Z DNA junction[31] suggests that this is not the case. In addition, we did not observe the re-reemergence of these peaks at higher concentrations of Zα which would be expected as the complex becomes more stable in the case of intermediate exchange."

- For example, AUC data on h43 would suggest a 4:1 complex being formed. However, the current Z-alpha binding site (Figure 3b) only spans 7 base pairs, coupled with the fact that a given protein binding site spans 5 base pairs, wouldn't this suggest that only two protein molecules can bind? Could there be 2 binding sites spanning bases 1-5 and 6-10 instead? Or is there only one binding site with non-specific binding of 2 additional proteins? Please clarify.

-Za can fit onto smaller stretches of RNA (4 base pairs, 3 in the case of a helix end) depending on the conformation adopted. We have confirmed this finding by measuring NMR on the A-Z 1 RNA control, which shows that Za only targets a stretch of 7 base pairs. This is in line with previous work on the DNA version of this control RNA which showed that 4 Za proteins bind to a stretch of 7 base pairs (Lee, Y. M. et al. NMR investigation on the DNA binding and B-Z transition pathway of the Zα domain of human ADAR1. Biophys. Chem. (2013). doi:10.1016/j.bpc.2012.12.002). As such, we do not

believe that the AUC and NMR results are in contradiction with each other. We have adjusted the text (page 11) to clarify this and take the new data into account.

- Similar arguments can also be applied to the H25 construct. The current binding site only has 5 base pairs, while the AUC data suggests a 1:4 complex. Could there be 2 binding sites instead from base pairs 3-7 and 8-12?

-Upon re-inspection of the titration of H25, we realized that we cannot confirm whether the peak for G4 disappears into the noise (indicating destabilization by Za), or if it simply coalesces with G28. Since the base-paired U27 does not disappear, this supports the latter explanation, and as such, the actual Za binding region now includes the lower half of the stem as well, and agrees with our AUC data. However, we have adjusted the text to reflect this uncertainty (page 12).

• Also, for the H66 construct, could there be 2 binding sites from 3-7 and 8-12? Given that the helix destabilizing region actually has many pyrimidine-purine steps conducive for Z-form formation, could 2 protein molecules not bind in the middle of the RNA?

- While we cannot rule out the possibly of intermediate exchange, we are confident that it is not, as detailed in our argument above.

Minor comments

- Potential typo in sentence 1 "… adenosine deaminase that acts on RNA 1 (ADAR1) …", Should this be changed to "… adenosine deaminase that acts on RNA (ADAR1) …"?

-We deleted the 1.

- It would be instructive to add amino acid residue numbering in Figure 1a, to enable readers to gauge the size of the domains and linkers.

-We have adjusted Figure 1 to take this suggestion into account.

- Does A to I editing by ADAR1 in Alu repeats occur in non CpG regions? It would be instructive to clarify this somewhere in the introduction.

-While Za binds to CpG regions due to their ability to easily adopt the Z-conformation, A-to-I editing is not carried out in such regions. Alu elements also contain very little CpG repeats, which is why we wondered whether Za could bind them.

- It would be instructive to show interactions formed by Lys169 with CpG RNA in Figure 1c, and position of residue 141 in Figure 6c, as these have key interactions with RNA/show large CSPs on RNA binding.

-We have adjusted Figure 1 and 6 to take these suggestions into account.

- Methods section: Circular Dichroism– “A control experiment was run to ensure that the absorbance of Zα alone in the 220-320 nm range at the same concentrations was minimal (Supplementary Figure S2)” - Examination of Supplementary Figure S2 suggests that absorbance of Z☐ alone does change from 220-250nm by an amount comparable to changes in absorbance in Z☐+RNA samples – please clarify. The authors should also add y and x axes labels to Figure S2.

-x and y axis labels were added.

-We have altered the following sentence: “A control experiment was run to ensure that the absorbance of Zα alone in the <u>**220**</u>-320 nm range at the same concentrations was minimal (Supplementary Figure S2)”

To this: “Control experiments were run to ensure that the absorbance of Zα and $Za_{Tyr177Ala}$ alone in the **250**-320 nm range **(the range which is reports on RNA secondary structure)** at the same concentrations was minimal (Supplementary Figure S2)”

- Methods section: ITC experiments – The authors should provide the two-site equation to used to fit ITC data in Figure 5a, in addition to mentioning how errors in Table 1 were computed.

-The ITC data was fit using Origin, which we now mention in the methods section, and also have added a reference to the ITC methods section that describes ITC fitting and analysis in detail.

- Methods section: Analytical centrifugation – Please provide a reference that describes fitting of the data.

-Added the following reference to the AUC methods section: Schuck, P. Size-distribution analysis of macromolecules by sedimentation velocity ultracentrifugation and Lamm equation modeling. *Biophys. J.* (2000). doi:10.1016/S0006-3495(00)76713-0.

- Methods section: NMR experiments – “The 2D 1H-1H NOESY recorded for H66 E. coli …” – Should this be changed to H66 H. sapiens?

-fixed.

- Methods section: Kd fitting for titration points – The authors should explain how they obtained the factor of 0.2 in front of the 15N chemical shift differences in the formula for computing the CSP. Should the factor not be □H/□N instead, where □ is the gyromagnetic ratio? The authors should also provide a derivation for the equation for estimating Kds from CSPs and mention any assumptions involved in its derivation, or refer to a prior study with this information.

-While some groups indeed use the ratio of the gyromagnetic ratios of 15N and 1H (~0.1), it is more common to use 0.2 because even the gyromagnetic ratio-corrected shift changes are typically different for 1H and 15N under the same perturbation.
-Added the following references: Montaville, P. *et al.* The PIP2 binding mode of the C2 domains of rabphilin-3A. *Protein Sci.* (2008)
And
Williamson, M. P. Using chemical shift perturbation to characterise ligand binding. Progress in Nuclear Magnetic Resonance Spectroscopy (2013)

- Figures 2b and c – Please use the same y-axis scales for the AluSx1 and h43 samples. This would facilitate easy comparison of the effects of the mutation.

-We have adjusted the axis scales

- Figure 2e – Please describe how the fractional decrease was computed in methods.

-Description added to Methods under the section: “Extent of Z-formation Calculation”.

- Figure S5 – For the panels on the right, please provide the x and y axes labels, in addition to explaining insets in the panels (please also do so for Figure 5e). The authors should also provide some sense of the errors in the AUC values and explain how band assignments were made. For example, for H25 E. Coli the major peak in the AUC data is at 36kDa, while the predicted weight for the assigned 1:4 complex is

39.1 kDa. Mentioning the complex stoichiometry next to the peak molecular weight in the AUC data would also be useful.

-We have added the requested axis labels to Figure S5 and Figure 5. We have added a description of potential errors in the AUC measurement to the Methods section.

- Figure 7 and "The binding of Zα to AluSx1Jo is more dynamic than for (CpG)3 RNA" section – Were the comparisons of kex between (CpG)3 DNA, (CpG)3 RNA and AluSx1Jo RNA made for similar populations of the bound and unbound forms? In all cases, exchange is between free and 2:1 (protein:RNA) complexes, right? Would be instructive to clarify this.

-All measurements were done at the same molar ratio of Za:RNA, which is why comparison is feasible. We have clarified this aspect in the text: "To answer this question, we acquired Carr-Purcell-Meiboom-Gill (CPMG) measurements at 2:1 Zα:AluSx1Jo (two Zα residues symmetrically bound around a single Z-step) and compared them to previous CPMG measurements on the $(CpG)_3$ DNA and RNA also measured at a 2:1 ratio of Zα:RNA [23]."

- Figure 5b - How were averages for R1, R1p and tau_corr computed for this figure. Can the set of numbers be used to get error bars for R1, R1p and tau_corr? Also, the authors should comment on why is correlation time for the 4:1 complex is less than that for the 2:1 complex, while the expectation based on the stoichiometry would suggest otherwise?

-The values were determined by averaging the residue specific R1, R1rho, and tauC values over the folded portion of Za. We cannot compute an error *per se* as it would not be a true error, but instead only reflect the variation across the residues.
We added the following comment to the Figure legend of Figure 5: "Average (of the corresponding residue-specific values) longitudinal (R_1), and rotating-frame ^{15}N NMR relaxation rates ($R_{1\rho}$), and the extracted effective overall correlation times (τ_{corr}) are plotted versus the molar ratio of Zα:AluSx1Jo in the experiment (residue-specific R_1, $R_{1\rho}$, and τ_{corr} are shown in Supplementary Figure 9)".
The X-axis is the molar ratio of Za:RNA, not the complex size. We have now added an x-axis title to make this more clear. We have also adjusted the following sentence: "These tumbling times were consistent with a 2:1 Zα:AluSx1Jo complex being formed **at a 4:1 and 2:1** protein:RNA molar ratio, an intermediate complex between 2:1 and 1:1 at a 1:1 molar ratio of protein:RNA and a 1:1 complex at 1:2 protein:RNA."

- Results: “A-Z junction adoption in AluSx1Jo, H66, h43, and H25 involves destabilization of adjacent A-RNA” section, Page 11 – “For H25, Zα appeared to target the region in the middle of the stem which contained only one (YpR) step and two G.G base pairs (Figure 3d).” – should this be G.A mispairs instead of G.G?

-This was a typo and is now fixed.

- Results: “Zα binds cooperatively to AluSx1Jo and h43 in the ~500 nM–2 μM range” section – “These tumbling times were consistent with a 2:1 Zα:AluSx1Jo complex being formed at a 2:1 protein:RNA molar ratio, a 1:1 complex at 1:2 protein:RNA, and an intermediate complex between 2:1 and 1:1 at a 1:1 molar ratio of protein:RNA” – I would suggest re-wording this sentence to – “These tumbling times were consistent with a 2:1 Zα:AluSx1Jo complex being formed at a 2:1 protein:RNA molar ratio, an intermediate complex between 2:1 and 1:1 at a 1:1 molar ratio of protein:RNA and a 1:1 complex at 1:2 protein:RNA”, for ease of readability.

-We agree with this suggestion. Text has been replaced.

Reviewer 2

This paper reports on biophysical study of the A-Z junction formation of various RNA induced by Za protein. The authors proposed that Za proteins bind to a CG-rich region in the non-CG-repeat RNA via novel mechanism which combines the CG-repeat DNA binding model and B-Z junction formation model. The results are of interesting and the experimental data and controls look convincing. Therefore, I recommend publication almost in the present form, but would suggest that the authors revised as considering the following:

1) The authors estimated the relative Z-contents by Ez from CD data and the Z-RNA forming and A-Z junction base-pairs by imino proton spectra. These approaches are of interest and could be applicated in other study of the nucleic acid-protein interactions. The authors determined the molar ratios of Za:RNA complexes by AUC. Generally, two Za molecules span 4~6 base-pairs via binding to each strand of RNA). For example, the Za:RNA complex with 4:1 molar ratio contains at least 8-bp Z-RNA (possibly 9~10 bp).

The contents of Z-RNA base-pair might be correlate with Ez value. Thus, I suggest the -authors discuss more the correlation between these parameters.

-We have added the following text to the discussion: "We attempted to correlate the calculated E_Z scores to other factors in our tested RNA fragments, such as the number and identity of dinucleotide steps within the RNA. However, the only significant correlations to come out of this analysis were to RNA length and the number of non-canonical base pairs. This highlights the complexity in the factors which contribute to the ability of a particular RNA to adopt the Z-conformation, of which our simple analysis was unable to uncover."

2) In addition, in Figure 3, when the Za binding region is determined, the Za:RNA molar ratio and Ez value are should be considered. In Discuss section, if you mention that, the reader can understand your concept more easily.
-We have adjusted the discussion section to now discuss this point.

3) I cannot find the AUC data for the AluSx1Jo RNA in Figure S5.
-We have measured this and it is now included in Figure S5. It agrees with our NMR data of a 2:1 Za:RNA complex.

4) When you mentioned Z-DNA binding model, the original paper about this model (Kang et al., JACS 131 (2009), 11485.) should be sited.
-We now cite this paper in the proper places.

Reviewer 3

Nichols and coworkers investigate the interaction between the Z-DNA binding domain of ADAR1 (Zalpha) and non-CpG repeats of various RNAs, e.g. Alu and rRNA. The authors show by a variety of techniques that a broad range of RNA sequences bind to Zalpha adopting a Z-RNA conformation, and being accompanied by a simultaneous destabilization of neighboring A-form regions. The knowledge on Zalpha (and other) Z-RNA inducing protein domains is very scarce and only at the very beginning of grasping its importance. In addition, this study is carefully conducted, and the results based on a broad variety of experiments. Hence, I can recommend this manuscript for publication in Nature Commun, taking the below minor comments into consideration first.

i) In the last sentence of the abstract the authors highlight the implication of their findings in the role of Zalpha with respect the attack of a pathogen on a cell. Such a statement should be made with care because in the final conclusion section, it becomes evident that basically all implications for any role of Zalpha are still hypothetical, with no real evidence (yet), e.g. typical phrases used are "if/could/would/remains unclear …" etc. This does by no means diminish the importance of the findings, but the authors should simply stick to the facts, and not imply anything that has not been shown. The results themselves are important enough. Please adjust.

-We have adjusted the abstract.

ii) page 5, calculation of Ez; it is unclear where the factors 1.8, 0.718, and 1.109 in the given equation come from. Please explain.

-We have added the following phrase to the methods: “the prefactors were chosen so that the E_Z score of the $(CpG)_3$ RNA is equal to one.”

iii) page 7, fitting of NMR titrations: the CSP is calculated from the average of 1H and 15N chemical shift changes. Why? It would be much better to fit the CSP of 1H and 15N individually and take the average. Also the factor 0.2 is not explained. In this whole section no reference is given, please add. The second equation provided (obviously coming from a quadratic equation) should be derived/explained in more detail. Such CSPs go back to the 70ies, originally derived for UV measurements (same dependence as NMR shifts), and have been adopted subsequently, see e.g. Mitchell in J Chem Soc Dalton Trans (1979) 771-776, and then slightly adopted, e.g. in Helv Chim Acta (1995) 78 : 1112-1143 or JBIC (2000) 5 : 287-299.
-It is standard to combine the 1H and 15N CSPs into one effective shift change (see added references). While some groups indeed use the ratio of the gyromagnetic ratios of 15N and 1H (~0.1), it is more common to use 0.2 because even the gyromagnetic ratio-corrected shift changes are typically different for 1H and 15N under the same perturbation.
-We have cited the following references in the Methods which explain chemical shift fitting in detail: Montaville et al. (2007), *Protein Science*.
and
Williamson (2013), *Prog. In. Nuc. Mag. Res. Spec.*

Reviewer #1 (Remarks to the Author):

The authors of addressed my original comments and the manuscript is acceptable for publication in Nature Communication
